# Expression and Roles of the Immunoglobulin Superfamily Recognition Molecule Sidekick1 in Mouse Retina

**DOI:** 10.3389/fnmol.2018.00485

**Published:** 2019-01-09

**Authors:** Masahito Yamagata, Joshua R. Sanes

**Affiliations:** Department of Molecular and Cellular Biology, Center for Brain Science, Harvard University, Cambridge, MA, United States

**Keywords:** laminar specificity, synapse formation, retinal ganglion cell, amacrine cell, bipolar cell

## Abstract

Processes of >100 types of interneurons (bipolar and amacrine cells) and projection neurons (retinal ganglion cells, RGCs) form specific and stereotyped patterns of connections in the inner plexiform layer (IPL) of the mouse retina. Four closely related homophilic immunoglobulin superfamily recognition molecules (Sidekick [Sdk] 1, Sdk 2, Dscam, and DscamL1) have been shown to play roles in patterning neuronal arbors and connections in chick retina, and all but Sdk1 have been shown to play related roles in mice. Here, we compare patterns of Sdk1 and Sdk2 expression in mouse retina and use genetic methods to assess roles of Sdk1. In adult retina, 3 neuronal types express *sdk1* but not *sdk2* at detectable levels, 5 express *sdk2* but not *sdk1* and 3 express both. Patterns of gene expression and protein localization at or near synapses are established during the first postnatal week. Dendrites of amacrine cells and RGCs that express *sdk1* but not *sdk2* arborize in the same narrow stratum in the center of the IPL. In the absence of Sdk1, this laminar restriction is degraded. Overexpression of *sdk1* in developing cells that normally express *sdk2* reorients their dendrites to resemble those of endogenously Sdk1-positive cells, indicating that Sdk1 plays an instructive role in patterning the IPL. Sdk1 fails to affect arbors when introduced after they are mature, suggesting that it is required to form but not maintain laminar restrictions. The effect of ectopically expressed sdk1 requires the presence of endogenous Sdk1, suggesting that the effect requires homophilic interactions among Sdk1-positive neurites. Together with previous results on Sdk2, Dscam, DscamL1, as well as the related Contactins, our results support the idea that an elaborate immunoglobulin superfamily code plays a prominent role in establishing neural circuits in the retina by means of tightly regulated cell type-specific expression and homophilically restricted intercellular interactions.

## Introduction

Over the past decade, the mouse retina has emerged as a valuable model for investigating how “hard-wired” neural circuits are assembled. In one of its two synaptic layers, the inner plexiform layer (IPL), neurites of >50 types of interneurons (bipolar and amacrine cells) form synapses on dendrites of >40 types of output neurons (retinal ganglion cells, RGCs) during the first two postnatal weeks (reviewed in Sanes and Zipursky, 2010; Hoon et al., 2014). The specific and stereotyped patterns of these connections endow each RGC type with selective sensitivity to specific visual features, such as motion in a particular direction, edges, or color contrasts (reviewed in Masland, 2012; Sanes and Masland, 2015). Analysis of these circuits has implicated a variety of recognition molecules in the cell-cell interactions that establish them; they include members of the immunoglobulin and cadherin superfamilies, the semaphorins and plexins, and others (e.g., Fuerst et al., 2008, 2009, 2012; Matsuoka et al., 2011; Kay et al., 2012; Lefebvre et al., 2012; Sun et al., 2013; Duan et al., 2014, 2018; Krishnaswamy et al., 2015; Peng et al., 2017; Liu et al., 2018; reviewed in Zhang et al., 2017). Together, these studies demonstrate that numerous recognition molecules act together to pattern neural circuitry in the IPL. Since all of these molecules are also expressed by neuronal subsets throughout the brain, insights obtained in studies of the retina are likely to be relevant to the central nervous system generally.

In this study, we focus on retinal expression and roles of two closely related immunoglobulin superfamily recognition molecules that have been implicated in retinal development, Sidekick 1 and 2 (Sdk1 and Sdk2). We isolated the Sdks in a search for genes expressed by subsets of RGCs in the developing chick retina (Yamagata et al., 2002). We named them for the related *Sdk* gene in Drosophila, which was identified in a screen for genes that affect patterning of the fly eye (Nguyen et al., 1997) and was recently shown to be required for synaptic targeting of photoreceptors (Astigarraga et al., 2018). The Sdks are large (~250 kD), proteins, with six immunoglobulin domains, thirteen fibronectin repeats, a single transmembrane domain, and a cytoplasmic domain ending in a PDZ domain-binding motif. They are homophilic adhesion molecules (Yamagata et al., 2002; Hayashi et al., 2005; Yamagata and Sanes, 2008). Structural studies have shown that the immunoglobulin domains mediate homophilic adhesion, and defined critical residues required for adhesion *per se* and for homophilic specificity (Goodman et al., 2016; Tang et al., 2018). Their PDZ-binding motif binds scaffolding proteins of the MAGI family, an interaction that contributes to their concentration at synaptic sites (Yamagata and Sanes, 2010).

In chick retina, the Sdks are expressed by non-overlapping subsets of retinal neurons, and required for restriction of neuronal processes to specific strata within the IPL (Yamagata et al., 2002; Yamagata and Sanes, 2008). Their closest relatives, two *Dscams* (*Dscam and DscamL*) and six *contactins* (*Cntn1-6*) are also expressed by neuronal subsets in chick retina and play related roles, leading to the suggestion that they comprise an “immunoglobulin superfamily code” for laminar specificity (Yamagata and Sanes, 2008, 2012a).

Recently, we analyzed expression and roles of Sdk2 in mice (Krishnaswamy et al., 2015). We found that *sdk2* is expressed by restricted subsets of retinal neurons, including an unusual glutamatergic amacrine interneuron called VGlut3-positive amacrine cells (VG3-ACs) (Haverkamp and Wässle, 2004; Johnson et al., 2004; Grimes et al., 2011) and an RGC type called W3B, which has the unusual property of responding when the timing of the movement of a small object differs from that of the background, but not when they coincide (Kim et al., 2010, 2015; Zhang et al., 2012; Lee et al., 2014; Krishnaswamy et al., 2015). We showed that VG3-ACs synapse on W3B-RGCs, that VG3 input is essential for W3B-RGC function, that Sdk2 is required for restriction of VG3-AC and W3B-RGC processes to appropriate strata, and that the number and strength of functional connections between VG3-ACs and W3B-RGCs are dramatically reduced in the absence of Sdk2 (Krishnaswamy et al., 2015).

Here, we have analyzed expression and roles of Sdk1 in mouse retina. Confirming initial observations (Krishnaswamy et al., 2015), we show that *sdk1*, like *sdk2*, is expressed by a small number of specific interneuronal and RGC types. As in chick, types that express *sdk1* and *sdk2* are largely non-overlapping, but we also found three types that express both *sdks*. *Sdk1*, like *sdk2*, in mice and both *sdks* in chick, is expressed by interneurons and RGCs that arborize in the same strata, and neurites of these cells exhibit decreased laminar restriction in the absence of Sdk1. Finally, we use ectopic overexpression of *sdk1* in cells that normally express *sdk2* to demonstrate that it plays an instructive role in laminar targeting and that it does so by a homophilic mechanism.

## Materials and Methods

### Animals

Animals were used in accordance with NIH guidelines and protocols approved by Institutional Animal Use and Care Committee at Harvard University. Production of mouse lines by genome editing was performed in the Genome Modification Facility, Harvard University.

To generate the *sdk1*^*CG*^ allele, CreGFP was amplified from Addgene plasmid #13766, and inserted at the translational start site of the *Sdk1* gene using CRISPR/Cas9 nickase-mediated genome engineering (Ran et al., 2013; Wang et al., 2013). The targeting vector was modified from that reported previously (Krishnaswamy et al., 2015) by substituting CreGFP for CreER^T2^. The template DNA sequences to generate the sgRNAs used to enhance homologous recombination were CGGCATGGCCCGCGCCCGGC and GGTGGCGGGCGGCGGAGTCG (see Figure S1). Two sgRNAs, the circular targeting construct, and the synthesized Cas9 nickase mRNA were injected into the cytoplasm of fertilized eggs. The indel mutant *sdk1*^Δ*N*^ was obtained from the same injections; in these mice, the *sdk1* gene was altered, but CreGFP was not inserted.

The *sdk1*^*CE*^ (3xHA-tagged CreER) and *sdk2*^*CE*^ (6xMYC-tagged CreER) mouse lines were described previously (Krishnaswamy et al., 2015). To generate *sdk2*^*C*^, two sgRNAs were designed to target the junction between Cre and ER^T2^ in *sdk2*^*CE*^, and coinjected with Cas9 nickase mRNA into *sdk2*^*CE*/*CE*^ embryos. The template DNA sequences to generate the sgRNAs were GCTCTCATGTCTCCAGCAGA and GTCCCTGACGGCCGACCAGA.

To enable expression of *sdk1* or *sdk2* under Cre-dependent control, we generated three lines. A cassette encoding Venus and Sdk1, Venus and Sdk2, or Venus plus APEX2NES (ascorbic acid peroxidase with a nuclear localization signal), separated by tripleF2A (3 tandem repeats of foot-and-mouth disease 2A peptide sequence) was cloned into a Rosa26CAG-STOP- targeting vector (Yamagata and Sanes, 2012b) to generate Rosa-CAG-LOX-STOP-LOX-Venus-3F2A-Sdk1-WPRE-FRT-neo-FRT, Rosa-CAG-LOX-STOP-LOX-Venus-3F2A-Sdk2-WPRE-FRT-neo-FRT, or Rosa-CAG-LOX-STOP-LOX-Venus-3F2A-APEX2NES-WPRE-FRT-neo-FRT. We refer to these lines as *RC-sV-Sdk1, RC-sV-Sdk2*, and *RC-sV-A*, respectively. Homologous recombinants were selected in the V6.5 ES cell line and chimeras were generated. Germ-line chimeras were crossed to a Flp-expressing mouse (Rodríguez et al., 2000) to remove the FRT-neo-FRT sequence.

We used several Cre-dependent reporter lines interchangeably. *Thy-STOP-YFP15* (referred to as *STOP15*) expresses YFP in a Cre-dependent manner under Thy1 regulatory elements (Buffelli et al., 2003). *Ai14* expresses tdTomato in a Cre-dependent manner (Rosa26-CAG-lox-stop-lox-tdTomato) (Madisen et al., 2010). Rosa-CAG-Lox-STOP-LOX-ChR2(H134R)-tdTomato mice (*Ai27*) expresses cell surface-localized channelrhodopsin following excision of a stop cassette by Cre recombinase (Madisen et al., 2012). *Ai14* and *Ai27* lines were obtained from The Jackson Laboratory (Bar Harbor, ME). The Rosa-CAGS-LOX-CHERRY-LOX-GFP line (referred to as *RC-FrePe*) was obtained from S. Dymecki (Harvard University) (Dymecki et al., 2010). The Cre-dependent tdTomato line *Colstd* (Collagen-CAG-loxP-STOP-loxP-tdTomato-WPRE) in the type I collagen locus (Beard et al., 2006) was generated in the V6.5 ES cells, and a mouse line was established from germ-line chimeras.

The *JamB-CreER* line to label J-RGCs was described previously (Kim et al., 2008). *ChAT-cre* (Rossi et al., 2011) was from The Jackson laboratory. *DAT-cre* mice, in which cre is targeted to the endogenous *DAT* locus (Zhuang et al., 2005) was obtained from X. Zhuang (University of Chicago) via V. Murthy (Harvard University). *Gbx2-CreERT2-IRES-GFP* (Chen et al., 2009) was a generous gift from James Y. H. Li (University of Connecticut).

To induce recombination in *CreER*^*T*2^ reporter lines, animals were injected with tamoxifen as follows: P2 pups were injected with 0.5 mg tamoxifen (T5648, Sigma, St. Louis, MO) in 0.05 ml sunflower oil (S5007, Sigma). P24 animals were injected with 5 mg tamoxifen in 0.5 ml oil. In some cases, animals were injected with 1 mg tamoxifen in 0.1 ml sunflower oil at 24 and 48 h prior to sacrifice, which resulted in translocating CreER^T2^ protein to the nucleus, enhancing our ability to detect it.

### Plasmids, Transfection, and RT-PCR

The mouse Sdk1 (long form) cDNA in pCMVscript (Clontech, Mountain View, CA) was described previously (Yamagata and Sanes, 2008). A cDNA encoding the short form of mouse Sdk1 was modified from the long form cDNA. Each plasmid was transfected to 293T cells (ATCC, Manassas, VA) with DMRIE-C (Thermo-Fisher, Waltham, MA) as described previously (Yamagata and Sanes, 2012a).

**Figure 1:**
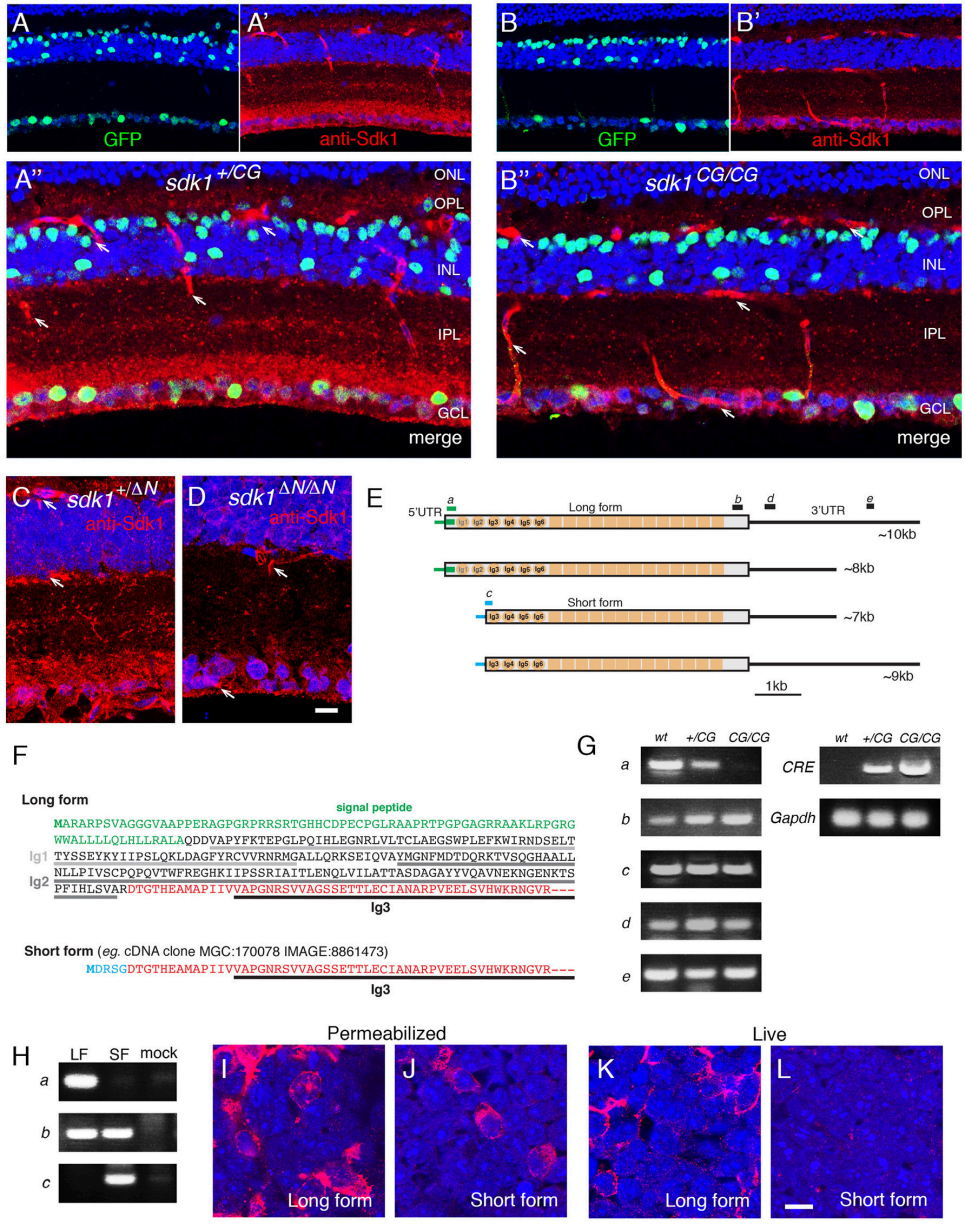
Evidence for an intracellular Sdk1 isoform. **(A,B)** Expression of *sdk1* and localization of Sdk1 protein in retina at P30. Sections from *sdk1*^+/*CG*^ **(A)** and *sdk1*^*CG*/*CG*^ **(B)** retina were stained with antibodies to GFP (green) and Sdk1 (red). In the *sdk1*^*CG*^ allele, CreGFP replaces the first coding exon; GFP reports on gene expression and Cre localizes GFP to the nucleus. Expression is similar in both *sdk1*^+/*CG*^ and *sdk1*^*CG*/*CG*^ retina, with positive cells in the ganglion cell layer (GCL) and INL but not the outer nuclear layer (ONL). Sdk1 is present in the inner plexiform layer (IPL) and GCL of *sdk1*^+/*CG*^ retina. IPL staining is absent in *sdk1*^*CG*/*CG*^ retina but some GCL staining persists. Staining of blood vessels (arrows) is non-specific. **(C,D)** In the *sdk1*^Δ*N*^ allele, the first 46 aa of Sdk1 are deleted (see Figure S1B). As in the *sdk1*^*CG*^ allele, Sdk1 is present in the IPL of *sdk*1^+/Δ*N*^ retina (C) and persists in the GCL of *sdk1*^Δ*N*/Δ*N*^ retina. Bar, 10μm for **(A–D)**. **(E)** *sdk1* transcripts, derived from entries in GenBank and the UCSC Genome browser, indicate occurrence of two 5′- and two 3′ sequences surrounding a common core. Letters a–e show positions of PCR primer pairs used in **(G)**. **(F)** 5′ coding sequence of the long and short forms. The short form lacks the signal peptide and the first two Ig domains (Ig1 and Ig2) critical for adhesion. Green, signal peptide; black, sequences in long form only; red, sequences in long and short forms; blue, sequences in short form only. Its first exon encodes five amino acids (MDRSG) absent from the long form. **(G)** Products amplified from wildtype (wt), *sdk1*^+/*CG*^ (+/CG), and *sdk1*^*CG*/*CG*^ (CG/CG) adult retinas by RT-PCR with primers shown in **(E)**, as well as *Cre* and *Gapdh* primers. The N-terminal region of the long form is not seen in *sdk1*^*CG*/*CG*^ retina. The results suggest that both the long and short protein coding regions can have either a long or short 3′ UTR. **(H)** Products amplified with primer sets a–c (see **E**) from 293T cells transfected with plasmids encoding Sdk1 long (LF) or short forms (SF) and untransfected controls (mock). As expected **(E)**, primer sets a and c distinguish long and short forms. **(I–L)** Sdk1 immunoreactivity of 293T cells transfected as in **(H)**. Cells were stained live **(I,J)** or after permeabilization **(K,L)**. Only the long form is accessible extracellularly. Bar, 10μm.

Total RNA from animals or cultured cells was isolated using illustra RNAspin Mini (GE Heathcare Life Sciences, Marlborough, MA), which uses deoxyribonuclease I to remove DNA. cDNA was generated with Superscript III (Thermo-Fisher/Invitrogen) using random or *sdk1* specific primers (CTCTATGATGGAAAGGAAGGCTC) for the short form, and treated with RNase H (Thermo-Fisher). Primer sequences and predicted sizes after PCR were as follows (see Figure 1 for location of each primer set).

- (161 bp): CCGGCGGGCGGCAAAGTTGAG, TGAGCACCAGGCGGTTCCCTTCC
- (243 bp): TCAAAGAAGAACGGAACCAGAT, CCGCTTCCAAGAGTTGTAGTAG
- (230 bp): AGTGATGGACAGATCAGGAGATA, ATGTCGGATTGGTGATGGTAAG
- (228 bp): AGGTATCTCCCTGGTGCAATA, GAGCCTCAAGTTGTCCTAAGATG
- (204 bp): GTAGGGACAGAATGGACACATC, CAGCTCACACAAGGAGGTAAG

EconoTaq plus green mixture (Lucigen, Middleton, WI) was used for PCR. PCR cycles were 94°C, 2 min; 42 cycles of 94°C, 30 s; 60°C, 30 s; 72°C, 1 min + 2 s extention; 72°C, 7 min, and 4°C.

Other primer sequences were as follows.

*Cre*: GCATTACCGGTCGATGCAACGAGTGATGAG, GAGTGAACGAACCTGGTCGAAATCAGTGCG

mouse *Gapdh*: TGAAGGTCGGTGTGAACGGATTTGGC, CATGTAGGCCATGAGGTCCACCAC.

### Antibodies

Antibodies used in this study were: rabbit monoclonal antibody to estrogen receptor α (ER) (Clone SP1, from Epitomics or Abcam, Cambridge, MA); goat anti-Myc (NB600-335, from Novus, Littleton, CO); rat anti-HA (3F10, from Roche Diagnostics Co., Indianapolis, IN); anti-Brn3a (clone, 5A3.2), rabbit anti-synapsin I (AB1543P), mouse anti-calretinin (clone, 6B8.2), sheep anti-tyrosine hydroxylase and goat anti-ChAT antibodies (AB144P) from Millipore (Billerica, MA); AP2 (clone, 3B5), SV2, and anti-synaptotagmin 2 (clone, ZNP1) from Developmental Studies Hybridoma Bank (Iowa City, IA); mouse anti-VGlut1 (clone, N28/9), mouse anti-pan-MAGUK (clone, N28/86), mouse anti-HCN4 (clone, N114.10), and mouse anti-Vesicular acetylcholine transporter (clone, N6/38) from NeuroMab (Davis, CA); mouse anti-protein kinase C- α (PKCα) (clone MC5) and rabbit anti-PKCα (P4334) from Sigma; rabbit anti-Opn4 from Thermo Fisher (PA1-780), mouse monoclonal antibody to neurofilament-H (phosphorylated) SMI-34 from Covance (Princeton, NJ); goat anti-Spp1 from R&D Systems (Minneapolis, MN). Rabbit antibody to Dab1 was a kind gift from Dr. Brian Howell (SUNY Upstate, Syracuse, NY). Chicken anti-GFP (Yamagata and Sanes, 2012b), rabbit anti-mCherry/RFP (Cai et al., 2013), and mouse antibodies to mouse Sdk1 or Sdk2 (Krishnaswamy et al., 2015) were generated in our laboratory. Nuclei were labeled with NeuroTrace 640 (ThermoFisher/Invitrogen). Secondary antibodies conjugated to dyes were from Jackson ImmunoResearch (West Grove, PA).

### Immunostaining and *in situ* Hybridization

For immunostaining, retinas were fixed with 4% (w/v) paraformaldehyde/PBS overnight at 4°C, sunk in 15%(w/v) and 30%(w/v) sucrose/PBS, and mounted in Tissue Freezing Medium (EM Sciences, Hatfield, PA). Sections were cut in a cryostat, permeabilized with 0.1% (w/v) TritonX-100/PBS for 5 min at room temperature, blocked with 5% (w/v) skim milk/PBS for 30 min at room temperature, incubated with appropriate antibodies overnight, rinsed, and incubated with appropriate secondary antibodies. After rinsing with PBS, sections were mounted in Fluoro-Gel (Electron Microscopy Sciences, Hatfield, PA, USA) and imaged with a Zeiss Meta510 confocal microscope (Oberkochen, Germany).

For double immunostaining with two mouse antibodies, we used the Zenon Horseradish Peroxidase Mouse IgG1 Labeling Kit (Life Technologies, Grand Island, NY) to label one of them, and detected reaction product with the TSA-Plus kit (Perkin-Elmer Life Sciences, Waltham, MA). For immunodetection of epitope-tagged CreER, cryosections were permeabilized in absolute methanol at −20°C overnight, treated with Image-iT FX signal enhancer (Life Technologies) by the manufacturer's protocol, and blocked with 5%(w/v) skim milk (BioRad, Hercules, CA) in PBS for 30 min at room temperature. The antibodies were diluted in Renoir Red diluent (BioCare Medical, Concord, CA), incubated at 4°C for 48 h, rinsed, and detected with secondary antibodies that had been preabsorbed with acetone powders prepared from mouse brain.

Whole-mount staining with antibodies was done as follows. Retinae were fixed with 4% (w/v) paraformaldehyde /PBS overnight at 4°C, and treated with 0.1% (w/v) TritonX-100/PBS for 30 min at 4°C. Tissues were then blocked with 1%(w/v) bovine serum albumin/PBS overnight, incubated with appropriate primary antibodies in 0.1% (w/v) TritonX-100/ 0.1%(w/v) bovine serum albumin/PBS for 48 h, rinsed with 0.1% (w/v) TritonX-100/PBS for 3 h, incubated with secondary antibodies in 0.1% (w/v) TritonX-100/ 0.1%(w/v) bovine serum albumin/PBS overnight, rinsed with 0.1% (w/v) TritonX-100/PBS overnight, mounted in glycerol-based VECTASHIELD (Vector labs, Burlingame, CA), placed on black nitrocellulose membranes (HABG01300, Millipore), and imaged with a Zeiss Meta510 confocal microscope. When anti-ER antibody was used, the retina was fixed with 4% (w/v) paraformaldehyde /PBS overnight at 4°C, and then placed in 0.1% (w/v) TritonX-100/PBS for 30 min at 4°C, 30%(v/v) methanol/0.1% (w/v) TritonX-100 for 30 min at 4°C, 50%(v/v) methanol/0.1% (w/v) TritonX-100 for 30 min at 4°C, 70%(v/v) methanol/0.1% (w/v) TritonX-100 for 30 min at 4°C, 100%(v/v) methanol overnight at −20°C, 70%(v/v) methanol/0.1% (w/v) TritonX-100 for 30 min at 4°C, 50%(v/v) methanol/0.1% (w/v) TritonX-100 for 30 min at 4°C, 30%(v/v) methanol/0.1% (w/v) TritonX-100 for 30 min at 4°C, and 0.1% (w/v) TritonX-100/PBS before blocking. Incubation with anti-ER (SP1) was done in the Renoir Red diluent supplemented with 0.1% (w/v) TritonX and 0.1%(w/v) bovine serum albumin, rinsed, and processed as described above.

Live staining of transfected 293T cells was performed as previously described (Goodman et al., 2016).

For *in situ* hybridization, riboprobes were synthesized from Sdk1cDNA using digoxigenin-labeled UTP (Roche) and hydrolyzed to around 500 bp. Probes were detected using horseradish peroxidase conjugated sheep antibodies to digoxigenin (Roche), followed by amplification with TSA-Plus system (Yamagata and Sanes, 2012a).

### Imaging and Statistical Analysis

Images were processed with Adobe Photoshop, and Image-J (Version 1.47d, Fiji). Position of spots were measured using Image-J. To generate the graphs in Figures 4F, 7G, 8C, 9D, 10J, cells were selected based on the clarity of their dendrites: we required that only a single cell be labeled in the field, and that a broad expanse of dendrite be visible in the field. Then, all GFP+ immunopositive spots that could be assigned to the cell's arbor were measured and counted. For Figure 5K, immunoreactive (calbindin+) puncta were counted.

To analyze variance of measured spots, the VAR.S function and *F*-tests were used in Microsoft Excel for Mac 2011 (version 14.3.1). “Variance” was calculated by VAR.S which uses this formula.

$$\begin{array}{l} \frac{{\sum \left(x_{m}-x\right)}^{2}}{\left(n-1\right)} \end{array}$$

where *x*_*m*_ is the sample mean and *n* is the sample size. To statistically compare two variances, s1 and s2, the *F*-test was performed using the following equation.

$$\begin{array}{l} \text{F}=s_{1}^{2}/s_{2}^{2} \end{array}$$

To generate box-plots, qplot function was employed in the ggplots2 package of R 3.4.4 for MacOS X GUI 1.70 (The R foundation, https://www.r-project.org/). ANOVA and Tukey *post-hoc* test were performed using R 3.4.4.

## Results

In a previous study (Krishnaswamy et al., 2015), we reported on the generation and use of knock-in mice in which a cDNA encoding a ligand -dependent Cre recombinase-human estrogen receptor (ER) fusion protein (CreER^T2^) was targeted to the first coding exon of the *sdk1* and *sdk2* genes, thereby disrupting the gene and generating null alleles (*sdk1*^*CE*^, *sdk2*^*CE*^). These lines allowed us to map cells that express *sdk1* or *sdk2* by staining for ER or for epitope tags appended to the CreER (HAtag for *sdk1*^*CE*^ and MYCtag for *sdk2*^*CE*^) or by crossing to a cre-dependent reporter. We showed that *sdk1* and *sdk2* are expressed in largely but not entirely non-overlapping subsets of retinal cells in mice, and analyzed *sdk2*^*CE*/*CE*^ mice to elucidate roles of Sdk2 in retinal circuitry. Here, we used these and newly generated alleles (Figure S1) to identify the cells that express *sdk1* and to analyze roles of Sdk1 in the retina.

### Multiple Sdk1 Isoforms

We used CRISPR/Cas9 technology to generate *Sdk1* alleles in which CreGFP replaced CreER (*sdk1*^*CG*^) or in which the first 46 amino acids were deleted without introduction of a reporter (*sdk1*^Δ*N*^). In characterizing these alleles, we stained retinal sections with anti-Sdk1. Immunoreactivity was present in the neuropil of the IPL of wild-type and heterozygous mice (*sdk1*^+/*CG*^ and *sdk1*^+/Δ*N*^) (Figures 1A,C), and this immunoreactivity was greatly attenuated in homozygotes (*sdk1*^*CG*/*CG*^ and *sdk1*^Δ*N*/Δ*N*^; Figures 1B,D). Surprisingly, however, immunoreactivity persisted in some somata in the ganglion cell layer (GCL). In exploring the origin of the residual staining, we noted that Kaufman et al. (2004) reported multiple *sdk1* mRNAs (6–10 kb) but only a single *sdk2* mRNA (~9 kb) by Northern analysis. Moreover, public databases report *sdk1* transcripts with at least two different 5′ ends and two different 3′ ends (Figure 1E). The two 5′ sequences encode different proteins. One, which we call the long form, is the previously documented Sdk1 protein, which has a signal peptide, 6 immunoglobulin (Ig) domains, 13 fibronectin type III repeats, a single-pass transmembrane, and a cytoplasmic domain. By contrast, the shorter form (e.g., IMAGE, 8861473) begins with a short exon encoding a putative initiation codon, but lacks the signal peptide and two Ig domains that are indispensable for homophilic adhesive activity of this molecule (Goodman et al., 2016) (Figure 1F). The *sdk1*^*CE*^, *sdk1*^*CG*^ *sdk1*^Δ*N*^ alleles are all predicted to inactivate the long but not the short form.

RT-PCR from retina confirmed that the long form is not expressed in *sdk1*^*CG*/*CG*^ mice, but the short form-specific exon, as well as sequences common to both forms, are present in both *sdk1*^+/*CG*^ and *sdk1*^*CG*/*CG*^ mice (Figure 1G). We also generated plasmids encoding long and short forms, expressed them in heterologous cells (Figure 1H) and stained the cells with antibodies to the Sdk1 ectodomain. Both forms were readily detected in permeabilized cells, but staining of live cells demonstrated that only the long form is present at the extracellular face of the cell surface (Figures 1I–L). These results indicate that the short form is present in retina, but confined to the cytoplasm and unable to participate in intercellular recognition.

### Sdk-Expressing Cells

The cells of the neural retina are divided into three cellular or “nuclear” layers, which are separated by two synaptic or “plexiform” layers. Photoreceptor somata occupy the outer nuclear layer (ONL), interneurons (horizontal, bipolar, and amacrine cells) and Müller glial cells occupy the inner nuclear layer (INL), and RGCs plus displaced amacrine cells occupy the GCL. Synapses of photoreceptors, horizontal cells and bipolar cell dendrites form the outer plexiform layer (OPL), and bipolar cell axons, amacrine cell processes and RGC dendrites synapse in the IPL.

**Figure 2:**
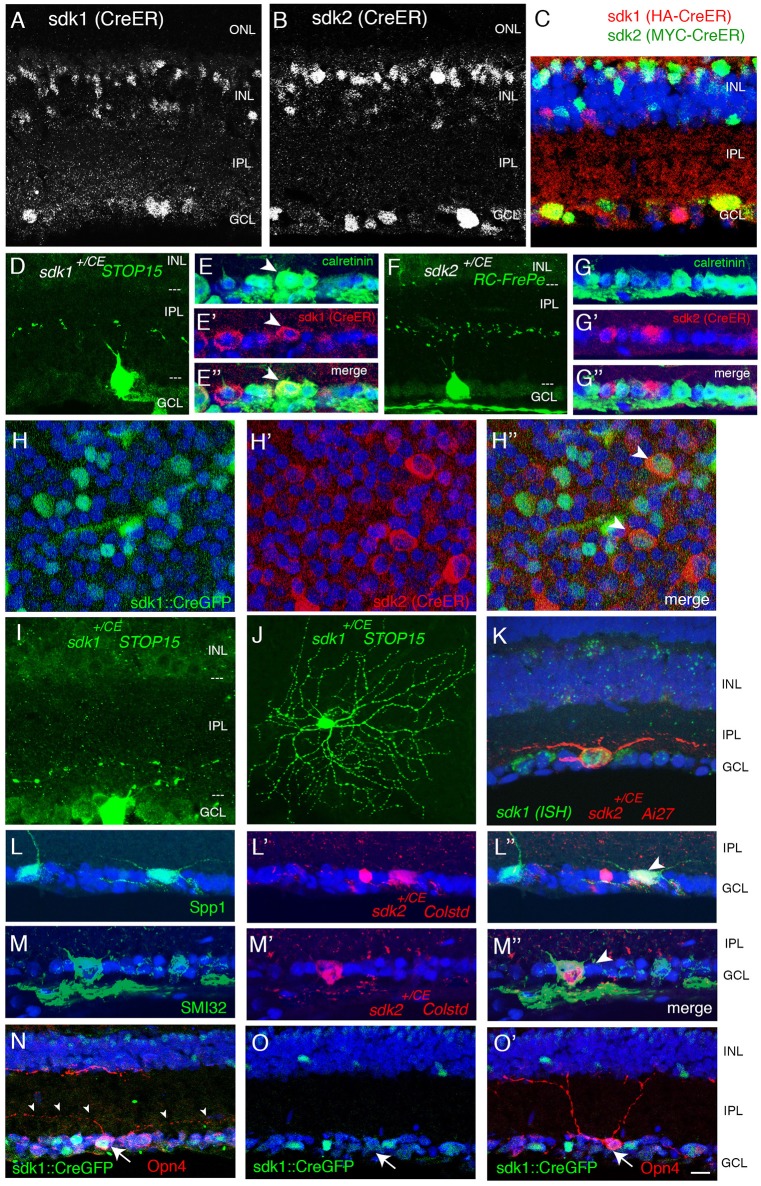
Retinal ganglion cells that express *sdk1* and/or *sdk2*. **(A,B)** Localization of CreER in *sdk1*^+/*CE*^ **(A)** and *sdk2*^+/*CE*^ **(B)** retina at P30. In the CE alleles, epitope-tagged CreER^T2^ replaces the first coding exon and is detected with anti-ER. Subsets of cells express CreER^T2^ in both lines. ONL, outer nuclear layer; OPL, outer plexiform layer; INL, inner nuclear layer; IPL, inner plexiform layer; GCL, ganglion cell layer. **(C)** Expression of CreER in *sdk1*^+/*CE*^;*sdk2*^+/*CE*^ retina The two alleles are distinguished by staining for their epitope tags, HA in *sdk1*^*CE*^ and MYC in *sdk2*^*CE*^. Some cell in the GCL and INL express both. **(D,F)** RGCs labeled in *sdk1*^+/*CE*^ **(D)** or *sdk2*^+/*CE*^ **(F)** mice mated to cre-dependent reporters (*STOP15* or *RC-FrePe*) following tamoxifen injection. Both *sdk1* and *sdk2* are expressed by RGCs with somata in the GCL and dendrites that arborize in S3 of the IPL, as shown with *sdk*^+/*CE*^; *reporter* mice. Arbors are narrow for sdk1+ RGCs and diffuse for sdk2^+^RGCs. **(E,G)** Many *sdk1*^+^RGCs are calretinin-positive (arrowheads in **E**), whereas sdk2+RGCs are calretinin-negative **(G)** as revealed by double staining with antibodies to calretinin and ER. **(H)** GCL of P30 *sdk1*^+/*CG*^ ; *sdk2*^+/*CE*^ retina stained as flat mounts with anti-GFP **(H)** and anti-ER **(H′)** to label cells expressing *sdk1* and *sdk2*, respectively. Most of the sdk+ cells express either *sdk1* or *sdk2*, but some express both (arrowhead). **(I,J)** Large RGCs with dendrites in S5 visualized in section **(I)** and flat mount **(J)** in *sdk1*^+/*CE*^ ;reporter (*STOP15*) mice following tamoxifen injection. Similar cells were observed in *sdk2*^+/*CE*^; *STOP15*. **(K)** Large RGC with dendrites in S5 visualized in section in a *sdk2*^+/*CE*^*; reporter* (*Ai27*) mouse (red). This RGC also expressed *sdk1*, as shown by *in situ* hybridization (green). **(L)** S5-laminating RGCs labeled in *sdk2*^+/*CE*^*; reporter* retina are stained with anti-Spp1 (osteopontin) which marks α-RGCs and M2 RGCs (arrowhead). **(M)** Some S5-laminating RGCs labeled in *sdk2*^+/*CE*^*;reporter* retina are stained by anti-neurofilament antibody SMI32, which labels α-RGCs but not M2-RGCs (arrowhead). **(N,O)** Some RGCs labeled in Sdk1^+/CG^ retina are stained by anti-Opn4 (melanopsin), which marks M1 and M2 RGCs but not α-RGCs. The double-labeled cell in **(N)** (arrow) bears dendrites in S5, identifying it as an M2 cell. The Sdk1-negative cell in **(O)** (arrow) has dendrites in S1, identifying it as an M2 cell. Bar, 20 μm for **(J)**, and 10 μm for others.

Using reporters and antibodies, as described above, we characterized retinal cells that expressed *sdk1* or *sdk2*. Neither *sdk1* or *sdk2* was detectably expressed by photoreceptors or Müller glial cells, but horizontal cells and some bipolar, amacrine cell, and RGC types expressed *sdk1* and/or *sdk2* (Figures 2A–C).

### Retinal Ganglion Cells

Over 40 RGC types have been described in mice (Baden et al., 2016; Bae et al., 2018; Rheaume et al., 2018). Of them, one is *sdk1*^+^*sdk2*^−^, one is *sdk1*^−^*sdk2*^+^ and two are *sdk1*^+^*sdk2*^+^.

The *sdk1*^+^*sdk2*^−^ RGC type has small somata and dendrites that ramify in a narrow sublamina at the center of S3 (Figure 2D). (By convention, the IPL is subdivided into 5 strata from S1 at the edge abutting the INL to S5 at the edge abutting the GCL.) This RGC type is calretinin-positive (Figure 2E). We provisionally call it the Sdk1+S3-RGC.

The *sdk1*^−^*sdk2*^+^ RGC type, which we characterized previously, is the W3B-RGC (Krishnaswamy et al., 2015). Like the Sdk1+S3-RGC, its soma is small, and its dendrites also ramify in S3. However, W3B dendrites are more diffuse than those of the Sdk1+S3-RGC, occupying most of the width of S3, and its somata are calretinin-negative (Figures 2F,G).

Both *sdk1*^+^*sdk2*^+^RGC types are large and have radial dendrites that stratify in S5 (Figures 2H–K). We identify one as the ONα-sustained RGC based on expression of Spp1(osteopontin) and SMI32 (Bleckert et al., 2014; Duan et al., 2015) (Figures 2L,M). The other is the M2 intrinsically photosensitive RGC based on its dendritic stratification, morphology and Opn4 (melanopsin) expression (Figures 2N,O).

### Amacrine Cells

There are ~60 amacrine cell types in mice (M. Laboulaye, W. Yan and J. R. Sanes, unpublished). Of them, two are *sdk1*^+^*sdk2*^−^ and two are *sdk1*^−^*sdk2*^+^. We found no amacrine cells that expressed both *sdk1* and *sdk2*.

**Figure 3:**
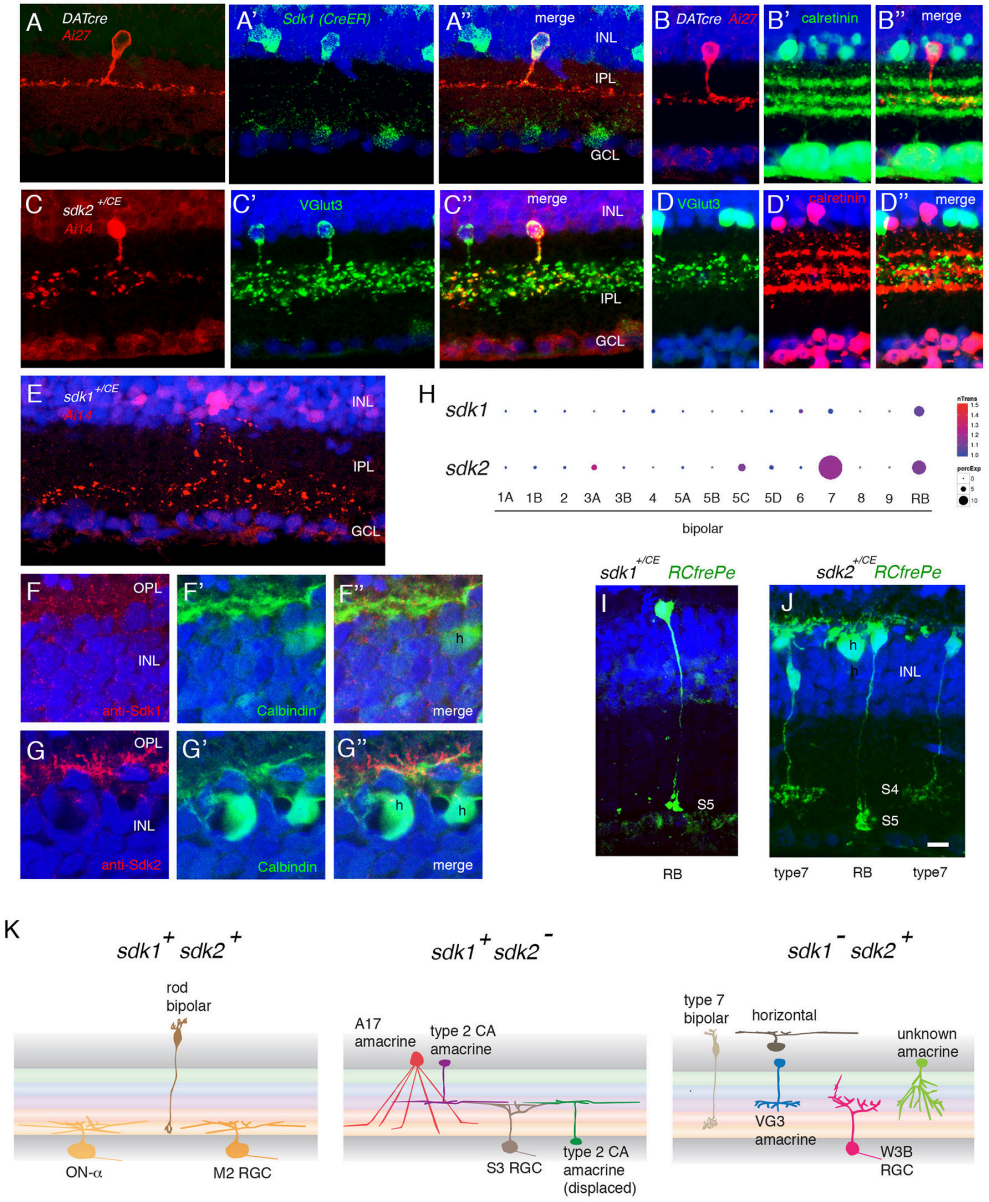
Interneurons that express *sdk1* or *sdk2*. **(A)** *Sdk1* is expressed by type 2 catecholaminergic amacrine (2AC) cells, labeled in the *DAT-cre* line. Image from a *DAT-cre;reporter* (tdTomato *Ai27*);*sdk1*^+/*CE*^ retina, stained for tdTomato **(A)** and ER **(A′)**; panel **(A″)** shows merge. **(B)** 2AC cells, labeled in *DAT-cre;reporter* (tdTomato *Ai27*) retina, stratify narrowly in S3, within the middle of three calretinin-positive sublaminae. **(C)** *Sdk2* is expressed by VG3 amacrine cells (*sdk1*^+/*CE*^; *reporter* (tdTomato *Ai14*); sectioned stained with anti-VGlut3). **(D)** VG3 amacrine cells (labeled with anti-VGlut3) arborize diffusely in S3 and are calretinin-negative. **(E)** Amacrine cell labeled in *sdk1*^+/*CE*^*;reporter* (*Ai14*) mouse following tamoxifen injection. The waterfall morphology is characteristic of A17 amacrine cells. **(F,G)** Horizontal cells (h) express Sdk2 **(F)** but not Sdk1 **(E)**. Sections were stained with anti-Sdk1 or anti-Sdk2 plus antibodies to calbindin, which marks horizontal cells. **(H)** Expression of *sdk1* and *sdk2* derived from single cell RNAseq data in Shekhar et al. (2016). RB, rod bipolar cells. *sdk2* is expressed by type 7 bipolar cells. Rod bipolar cells express both *sdk1* and *sdk2*. **(I)** Labeling of BCs in a *sdk1*^+/*CE*^*; reporter* (*RC-FrePe*) mouse. Bulbous terminals in S5 are indicative of RBs. **(J)** Labeling of BCs in a *sdk2*^+/*CE*^*; reporter* mouse. Broad axonal terminals in S4 are indicative of BC7. RBs are also labeled. A horizontal cell (h) is also labeled in this section. **(K)** Schematic of cell types that express *sdk1* and/or *sdk2*. Sketches summarize data from this figure, Figure 2 and Figure S2. Bar, 3 μm in **(E,F)**, 10 μm for others.

One of the two *sdk1*^+^*sdk2*^−^ amacrine types has processes that ramify in S3 (Figures 3A,B). Most if not all of these cells are calretinin-positive type 2 catecholaminergic amacrine cells (2CA-ACs), as demonstrated by use of the *DAT-cre* line (Figure 3A) (Contini et al., 2010; Knop et al., 2011). The 2CA-ACs are present in both the INL and the GCL. The *DAT-cre* driver preferentially labels the group in the INL (Figure 3A), whereas both sets are Gbx2-positive (see below). 2CA-ACs narrowly stratify at the center of S3 which corresponds to the middle of three calretinin-positive bands (Figure 3B).

The second sdk1^+^sdk2^−^ amacrine type is the A17 type, identified by the striking “waterfall” shape of its arbor (Figure 3E), and tentatively characterized by its expression of protein kinase Cα (Puthussery and Fletcher, 2007; Downie et al., 2009) (Figure S2A).

We showed previously that the predominant *sdk1*^−^*sdk2*^+^ type is the VGlut3-positive amacrine cell (VG3-AC), an unusual excitatory amacrine cell with processes in S3 (Krishnaswamy et al., 2015; see Figure 3C). VG3-ACs are calretinin-negative and arborize diffusely in S3 (Figure 3D). We also found a small number of a second type of *sdk2*^+^ amacrines that arborize in S3 but are distinct from VG3-ACs (Figures S2B,C). These were seldom encountered and have not been characterized further.

The *sdk1*^+^*sdk2*^−^ and *sdk1*^−^*sdk2*^+^ amacrine cells (VG3-AC and 2CA-AC) and RGCs (W3B-RGC and Sdk1+S3-RGC) are similar in that all four types have dendrites that arborize in S3. However, the fine details of their arbors differ. Our observation is consistent with our earlier observation that W3B-RGC is only weakly synaptically connected to 2CA-AC (Krishnaswamy et al., 2015), but appears to contrast an idea proposed by others (Brüggen et al., 2015) in that study, however, W3B-RGCs are not distinguished from related types.

Dendrites of the Sdk1+RGCs and the 2CA-ACs are larger in diameter and arborize in a narrow stratum in the center of S3. In contrast, dendrites of the Sdk2+ types (W3B-RGC and VG3-AC) are smaller in diameter and arborize more diffusely in S3. Sdk1^−^+2CA cells do innervate Sdk2+W3B-RGCs (Brüggen et al., 2015), but physiological analysis indicated that the connection is far weaker than that of Sdk2+VG3-ACs with W3B-RGCs (Krishnaswamy et al., 2015).

### Horizontal Cells

Horizontal cells express *sdk2* but not *sdk1* (Figures 3F,G). Strong punctate staining with anti-Sdk2 is seen in the outer plexiform layer (OPL), where photoreceptor synapses are abundant.

### Bipolar Cells

There are 15 types of bipolar cells in mouse retina (Shekhar et al., 2016). One is *sdk1*^−^*sdk2*^+^ and another is *sdk1*^+^*sdk2*^+^.

Our recent transcriptomic analysis of mouse bipolars demonstrated selective expression of *sdk2* in type 7 bipolar cells (Shekhar et al., 2016) (Figure 3H). Using reporters, we confirmed expression of *sdk2* in cells with axonal arbors arborizing in IPL sublamina S4, which is characteristic of type 7 bipolars (Figure 3I).

**Figure 4:**
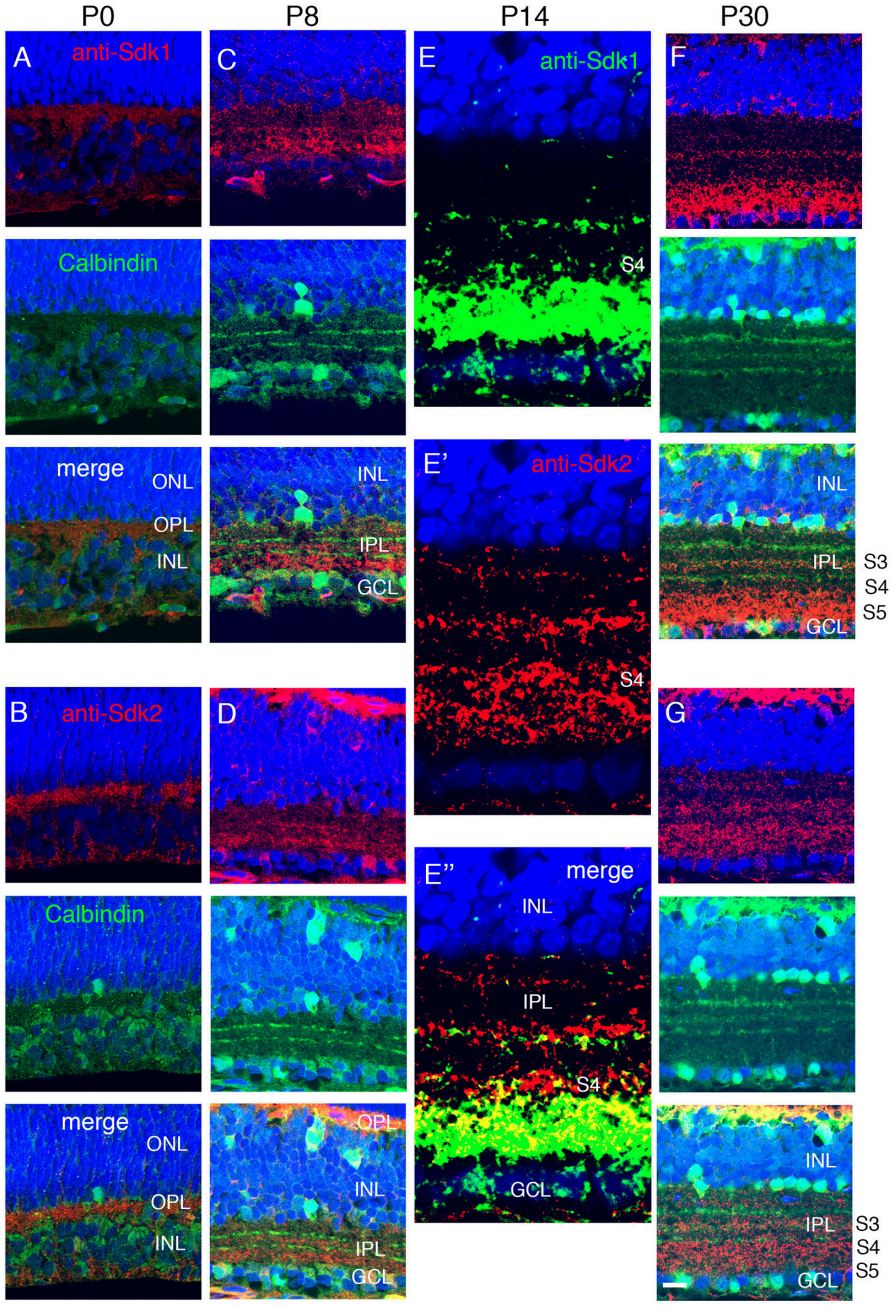
Localization of Sdk1 and Sdk2 during retinal development. Sections from P0 **(A,B)**, P8 **(C,D)**, P14 **(E)**, or P30 **(F,G)** mice were stained with anti-Sdk1 **(A,C,E,F)** or anti-Sdk2 **(B,D,E′,G)**. Sections in **(A–D,F,G)** were double stained with anti-calbindin, which labels three narrow strata in the IPL (see Figure 2). Section in **(E)** was doubly stained with anti-Sdk1 and anti-Sdk2. **(A,B)** Little Sdk1 immunoreactivity is present at P0 **(A)**, but Sdk2 is present in the OPL **(B)**. **(C,D)** At P8, Sdk1 and Sdk2 are both present in S3-5 of the IPL. **(E)** By P14, Sdk1, and Sdk2 are present in a narrow band in S3 and a broader band in S4,5. Sdk1+ and Sdk2+ immunoreactive puncta overlap in S4,5 but are distinct in S3. **(F,G)** Pattern at P30 is similar to that at P14. Bar, 8 μm for **(E)**, and 25 μm for others.

Both *sdk1* and *sdk2* are expressed by rod bipolar (RB) cells (Figures 3H–J). Axons of these cells ramify in S5, which is strongly stained with both anti-Sdk1 and anti-Sdk2 antibodies (Figures 4E–G).

Patterns of *sdk1* and *sdk2* expression in mouse retina are summarized in Figure 3K and Table 1.

**Table 1:** Cell types expressing *sdk1* and/or *sdk2*.

**Cell type**	**Class**	**Marker**	**IPL stratification**	**References**
***sdk1*****+** ***sdk2*****+**				
ON-α-sustained	RGC	Spp1+, SMI32+	S4-5	Bleckert et al., 2014; Krieger et al., 2017
M2	RGC	Opn4+	S4-5	Schmidt and Kofuji, 2009; Berson et al., 2010
Rod bipolar	Bipolar	PKCα+	S5	Greferath et al., 1990
***sdk1*****+** ***sdk2*****−**				
Sdk1+S3-RGC	RGC	Calretinin+	S3 narrow	Possibly 5to in Bae et al., 2018
2CA	Amacrine	Calretinin+ *DAT-cre+, Gbx2*+	S3 narrow	Contini et al., 2010; Knop et al., 2011
A17	Amacrine	PKCα**+**, calretinin−, Dab1−	S5 and others	Menger and Wässle, 2000; Puthussery and Fletcher, 2007; Downie et al., 2009
***sdk1*****−*****sdk2*****+**				
W3B	RGC	*TYW3*	S3 (+S1) broad	Kim et al., 2010; Zhang et al., 2012
VG3	Amacrine	VGlut3**+**, PKCα−, calretinin−, Dab1−	S3 broad	Haverkamp and Wässle, 2004; Johnson et al., 2004; Grimes et al., 2011
Type 7 bipolar	bipolar	GUS8.4GFP	between S4 and S5	Wässle et al., 2009; Shekhar et al., 2016
Unknown	amacrine	VGlut3−, PKCα−, Dab2−	S1-S4	N/A
Horizontal	horizontal	calbindin+	N/A	Pasteels et al., 1990

### Localization of Sdk1 an Sdk2 in Developing Mouse Retina

To ask when Sdk proteins appear during postnatal development, we stained retinas with antibodies to Sdk1 and Sdk2. Sdk2 was present in the OPL at P0, but neither Sdk1 nor Sdk2 was present at high levels in the IPL at this stage (Figures 4A,B). Both Sdk1 and Sdk2 were readily detectable within the IPL by P8 (Figures 4C,D). Both were present at highest levels in S3-5 by this time.

By P14, the staining pattern was similar to that in adults (Figures 4E–G). Both Sdks were present in S3 of the IPL, but in a non-overlapping distribution, with Sdk1 concentrated in a narrow stratum in the center of S3 and Sdk2 diffusely distributed throughout this sublamina. Both Sdk1 and Sdk2 were also present in S5, with greater overlap. Both were also present in S4, but with stronger staining for Sdk2 than Sdk1 (Figure 4E), likely reflecting the presence of Sdk2 but not Sdk1 in type 7 bipolar cells. These patterns of localization are consistent with the cell types expressing *sdk1* and/or *sdk2*, and suggest that Sdk proteins are concentrated at or near synapses, as shown previously in chicks (Yamagata et al., 2002).

### Lamination Defects in *sdk1* Mutant

Sdk1 mutants (*sdk1*^*CE*/*CE*^, *sdk1*^*CG*/*CG*^, and *sdk1*^Δ*NΔ*^^N^) are viable and fertile and no abnormalities were visible on inspection of the live animal or upon inspection of major organs following euthanasia and dissection. To assess retinal structure and molecular architecture in mutants, we stained sections with a panel of antibodies to 14 cell class-specific, cell type-specific and synaptic markers (Figure S3). In no cases did we detect differences in level or distribution of the marker between homozygotes and controls (wild types and heterozygotes).

In a previous study, we demonstrated a role for Sdk2 in the development of Sdk2-positive VG3-ACs and W3B-RGCs. Processes of both cell types arborize in S3, and VG3-ACs synapse on W3B-RGCs. In Sdk2 mutants, the arbors of these cells extend beyond S3 and the strength of VG3-W3B synapses is reduced by at least an order of magnitude (Krishnaswamy et al., 2015). We were not able to selectively label Sdk1+S3-RGCs to target them for recording, because our labeling methods preferentially marked 2CA-ACs and S5-arborizing RGCs in the ganglion cell layer. We therefore used histological methods to seek defects in Sdk1 mutants.

**Figure 5:**
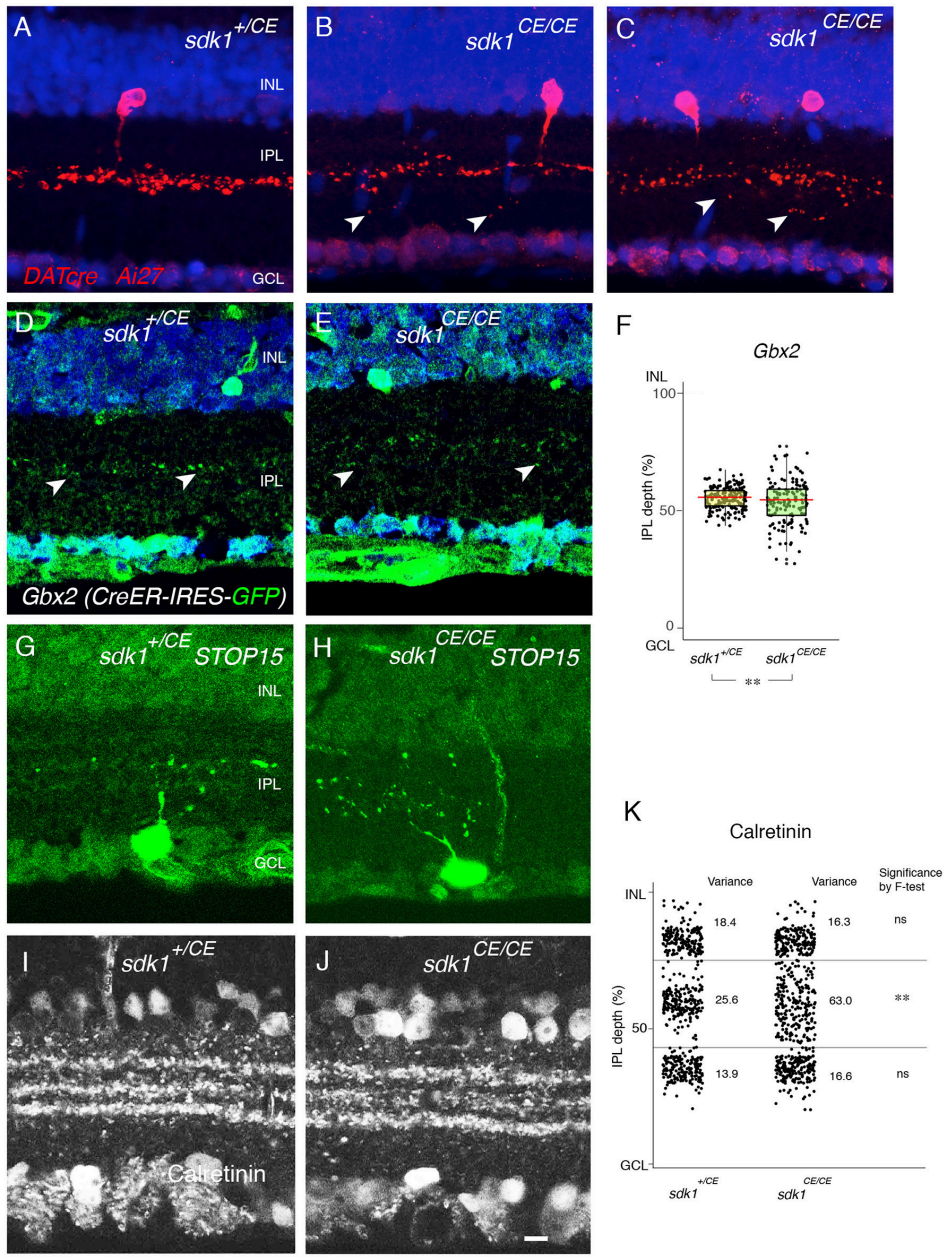
Loss of function phenotype in *sdk1* mutants. **(A–C)** Type II catechoraminergic amacrine (2CA) cells in INL labeled in *DATcre;*reporter (*Ai27*) mice in the presence **(A)** or absence of *sdk1* **(B,C)**. In homozygotes, stratification of 2CA cells is diffuse, associated with occasional sprouting (arrows). **(D,E)** Stratification of 2CA dendrites in S3 of *Gbx2-CreERT2-IRES-GFP*; this line labels 2CA cells in both INL and GCL in *sdk1*^+/*CE*^ **(D)** or *sdk1*^*CE*/*CE*^ **(E)**. **(F)** Position of GFP-positive dendritic segments plotted from micrographs such as in **(D)** (*n* = 6 areas from 3 animals) and **(E)** (*n* = 6 areas from 3 animals). The laminar position of GFP+ spots were measured and box-plotted **(F)**. Box-plots show upper and lower quartiles (box), median (horizontal line in the box), and the highest and lowest value excluding outliers (lines). The variance score is 2.0 for *sdk1*^+/*CE*^ and 8.4 for *sdk1*^*CE*/*CE*^. *F*-test for the variation is significant (**F < 0.0001). **(G,H)** Cells in the GCL and their dendritic arbors were labeled by crossing *sdk1*^+/*CE*^ **(D)** or *sdk1*^*CE*/*CE*^ **(E)** to the *STOP15* reporter. Dendritic arbors in homozygotes are more diffusely distributed in the IPL than that in heterozygotes. **(I–J)** Localization of calretinin in *sdk1*^+/*CE*^ **(I)** or *sdk1*^*CE*/*CE*^ **(J)** mice. **(K)** The position of the calretinin+ spots were measured from micrographs such as those in **(I)** (*n* = 3 areas from 3 animals) and **(J)** (*n* = 3 areas from 3 animals), and box-plotted as in **(F)**. *F*-test for the variation is significant (^**^F < 0.0001) for the middle band. Variation of the outer and inner calretinin-positive bands, corresponding to sdk-negative processes including starburst amacrine cells, is unaltered. Bar, 10μm.

First, we examined 2CA-ACs labeled in the *DAT-cre* line. In heterozygotes (*DAT-cre*; *reporter*; s*dk1*^+/*CE*^) dendrites were confined to a narrow stratum in the center of S3, as shown above (Figures 3A,B). In the absence of sdk1, however (*DAT-cre*; *reporter*; s*dk1*^*CE*/*CE*^), dendrites sprouted beyond their laminar boundary (Figures 5A–C). Similar results were obtained using a *Gbx2* (*Gbx2-CreER*^*T*2^*-IRES-GFP*) mouse line (Chen et al., 2009) which labels 2CA-ACs in both INL and GCL. 2CA-ACs sharply stratify in S3 (Figure 5D,E). To quantify the effect of Sdk1 deletion on 2CA-AC arbors, we plotted the laminar position of GFP-labeled dendritic segments, which appeared as spots in the micrographs. We then calculated the variance in position as an approximation of the diffuseness of the arbors within S3 (see Materials and Methods). The “Variance” scores from each dataset are shown in Figure 5F. An *F*-test demonstrated that the arbors were significantly more diffuse in *sdk1* mutant homozygotes than in heterozygotes.

To analyze arbors of the Sdk1+ RGCs that laminate in S3, we compared *STOP15*; s*dk1*^+/*CE*^) and *STOP15*;s*dk1*^*CE*/*CE*^ mice following administration of tamoxifen at P0 (Figures 5G,H). Cells were labeled sparsely in this genotype, making satisfactory quantification infeasible, but multiple examples showed that dendrites of Sdk1+S3-RGCs sprouted beyond their laminar boundary in mutants. However, both Sdk1-postitive 2CA-ACs and Sdk1+S3-RGCs are characterized by their expression of calretinin (Figures 2D,E, 3A,B), so we quantified the localization of calretinin in the IPL. The central band, which contains dendrites of Sdk1+S3-RGCs and 2CA-ACs, was more diffuse in the absence of Sdk1 than in its presence. The effect was specific in that the inner and outer bands, which contain dendrites of Sdk1-negative starburst amacrine cells, was not affected (Figures 5I–K). Together, these results suggest that neuronal processes of *sdk1*-expressing RGCs and amacrine cells in S3 exhibit decreased laminar restriction in the absence of Sdk1.

### Lamination Defects in sdk1/sdk2 Double Mutants

**Figure 6:**
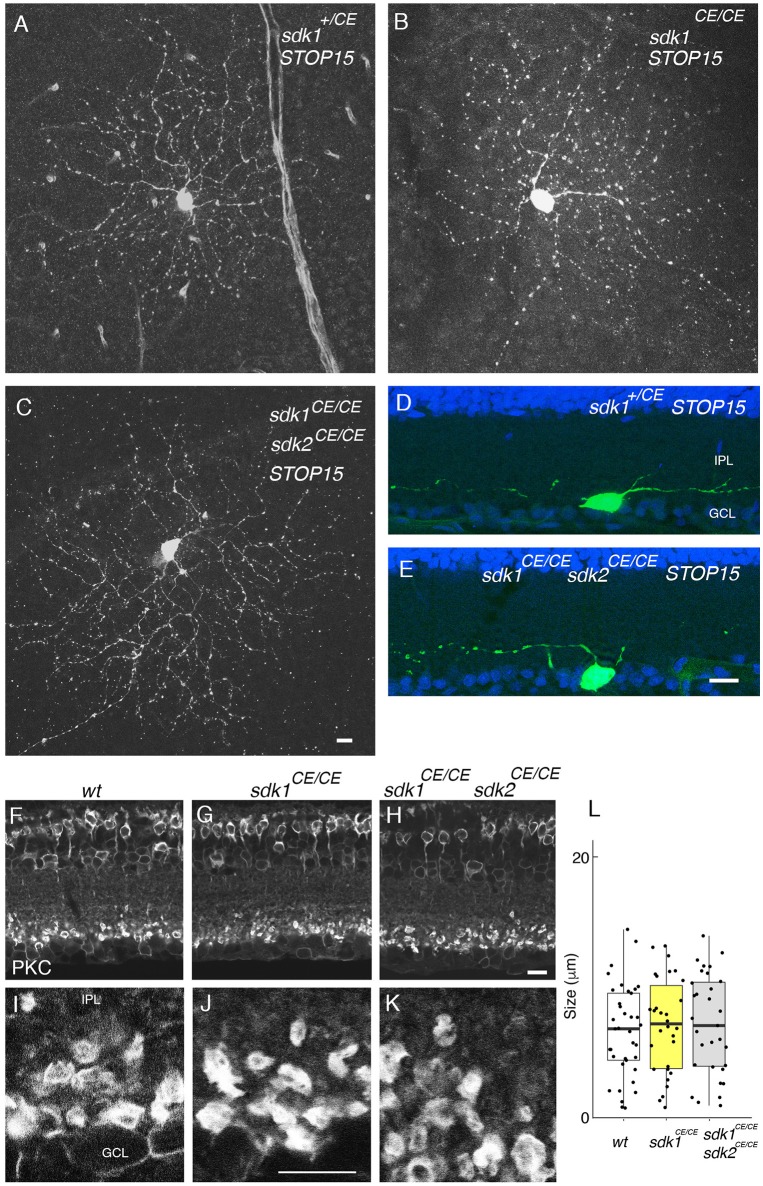
Phenotype in *sdk1 sdk2* double knock-out retina. **(A–E)** Large RGCs with dendrites in S5, viewed in flat mounts of tamoxifen-injected *sdk1*^+/*CE*^ **(A)**, *sdk1*^*CE*/*CE*^ **(B)**, or *sdk1*^*CE*/*CE*^ *sdk2*^*CE*/*CE*^ **(C)** mice or in sections of *sdk1*^+/*CE*^ **(D)** or *sdk1*^*CE*/*CE*^ *sdk2*^*CE*/*CE*^ **(E)** mice. Cell were visualized with the *STOP15* reporter. **(F–L)** Rod bipolar terminals in S5 labeled with anti-PKCα in wildtype **(F,I)** *sdk1*^*CE*/*CE*^ **(G,J)** or *sdk1*^*CE*/*CE*^ *sdk2*^*CE*/*CE*^ **(H,K)** mice. Overall stratification was not affected in *sdk1*^*CE*/*CE*^ or *sdk1*^*CE*/*CE*^ *sdk2*^*CE*/*CE*^ **(F–H)**. The circumference of PKCα-stained terminals **(J–K)** was measured, and plotted **(I)** (*n* = 31–38 from one animal each). Differences among genotypes were not significant by one-way ANOVA [Pr(>*F*) = 0.81; *p* > 0.8 by Tukey *post-hoc* test]. Bar, 10 μm.

We labeled ONα RGCs and rod bipolar cells, which express both *sdk1* and *sdk2*, in *sdk1*, and *sdk2* single mutants and in *sdk1sdk2* double mutants. Although the number of cells analyzed was insufficient for detailed quantification, we detected no obvious defects in the dendritic arbors of the ONα RGCs (Figures 6A–E). Likewise, the laminar position and size of rod bipolar terminals was unaffected in *sdk1sdk2* double mutants (Figures 6F–L). We also asked whether defects in VG3-ACs, which require Sdk2 for laminar restriction (Krishnaswamy et al., 2015) were more severe in double mutants, and found that they were not (Figure S4).

### Sdk1 Acts Homophilically to Pattern Dendrites

**Figure 7:**
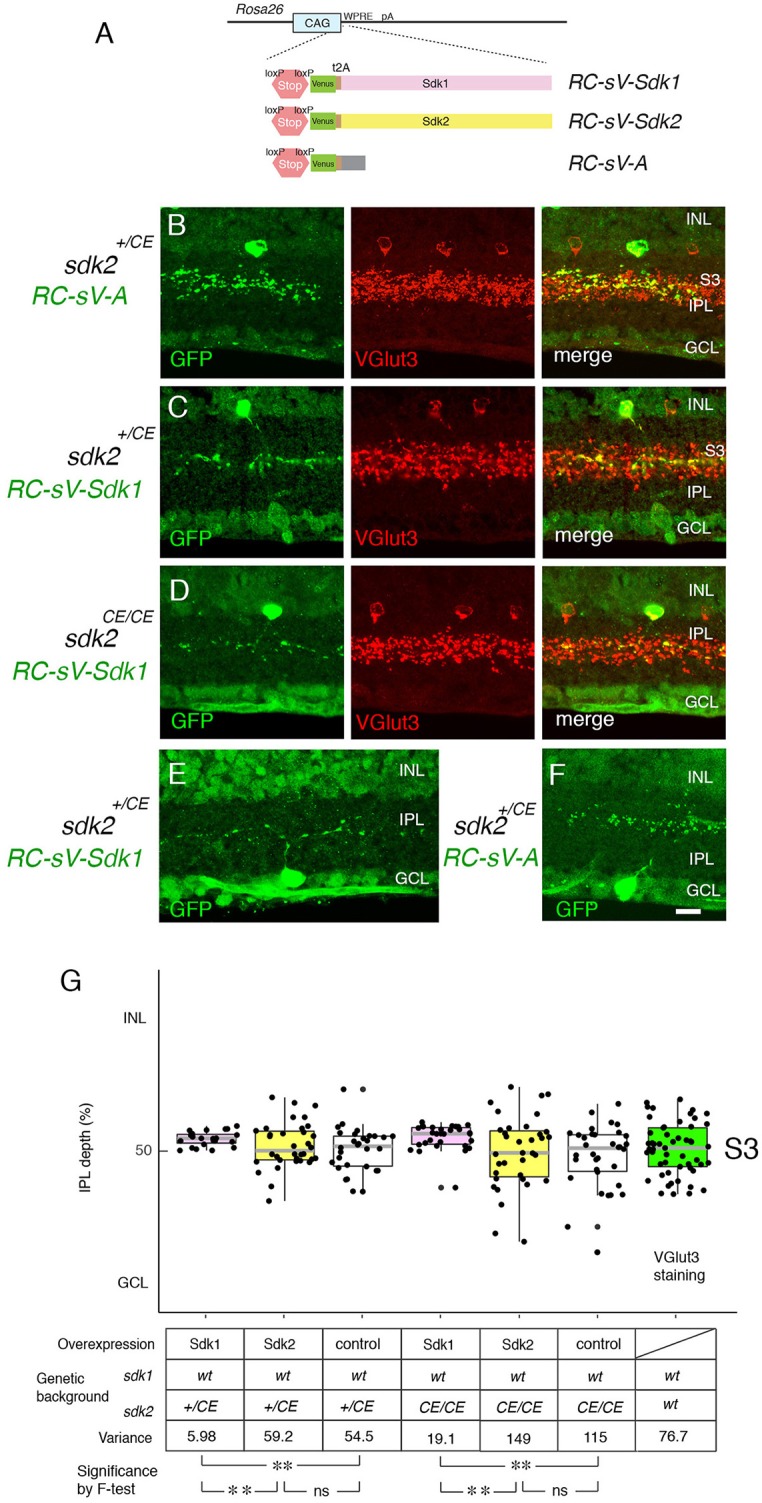
Overexpression of Sdk1 patterns dendrites. **(A)** Design of *RC-sV-Sdk1, RC-sV-Sdk2*, and *RC-sV-A* mouse lines. By crossing *RC-sV-Sdk1* and *RC-sV-Sdk2* to appropriate *Cre* driver lines, Sdk1 and Sdk2 can be overexpressed. The Sdk proteins are coexpressed with Venus (GFP) via a self-cleavable F2A sequence. *RC-sV-A* expresses Venus but not Sdk following Cre-mediated recombination. **(B–D)** VG3 cells labeled in *sdk2*^+/*CE*^*; RC-sV-A* mice exhibited characteristic morphology **(B)**. Expression of *sdk1* in *sdk2*^+/*CE*^*; RC-sV-Sdk1* **(C)** or *sdk2*^*CE*/*CE*^*; RC-sV-Sdk1* mice **(D)** resulted in the VG3 ACs acquiring a narrow arbor similar to that of Sdk1+ CA2 ACs. Pups were injected with tamoxifen at P2 dissected at P32, and stained with antibodies to GFP and VGlut3. **(E,F)** W3B RGCs labeled in *sdk2*^+/*CE*^*;RC-sV-Sdk1* **(E)** or *sdk2*^+/*CE*^*;RC-sV-A* **(F)** mice. Expression of *sdk1*resulted in the W3B RGCs acquiring a narrow dendritic arbor similar to that of the Sdk1+RGCs shown in Figure 2. Pups were injected with tamoxifen at P2, and dissected at P32. Bar indicates 10μm for **(A–F)**. **(G)** Laminar position of GFP+ spots from indicated genotypes was measured in micrographs such as those shown in **(B–D)**, and plotted as in Figure 5 (*n* = 5–6 neurons from each of 3 animals). Plot at far right derived from VGlut3 staining. Variance scores are shown under the graph. Statistical significance of variance by *F*-test: ns, *F* > 0.1; **F < 0.001.

To probe roles of Sdks further, we generated lines in which expression of *sdk1* or *sdk2*, along with a green fluorescent protein (Venus), required cre-mediated excision of a STOP cassette (Figure 7A). We crossed these mice to the *sdk2*^+/*CE*^ line and delivered tamoxifen at P2, thereby expressing *sdk1* or *sdk2* plus Venus in a sparse subset of cells that normally express *sdk2*. Ectopic expression of *sdk1* in W3B-RGCs or VG3-ACs led to formation of narrowly stratified arbors, similar to those of Sdk1-positive Sdk1+S3-RGCs or 2CA-ACs, respectively (Figures 7C,E,G). In contrast, the arbors were unperturbed by expression of *sdk2* plus Venus or of Venus alone (Figures 7B,F,G).

As noted above, satisfactory quantification was infeasible for RGCs, but we used analysis of variance to quantify these effects for VG3-ACs, demonstrating that they were highly significant. We also showed that Sdk1 acted similarly in a Sdk2 mutant background (*sdk2*^*CE*/*CE*^ ) (Figures 7D,G). Thus, Sdk1 can pattern dendritic arbors in cells that are normally *sdk1*-negative.

**Figure 8:**
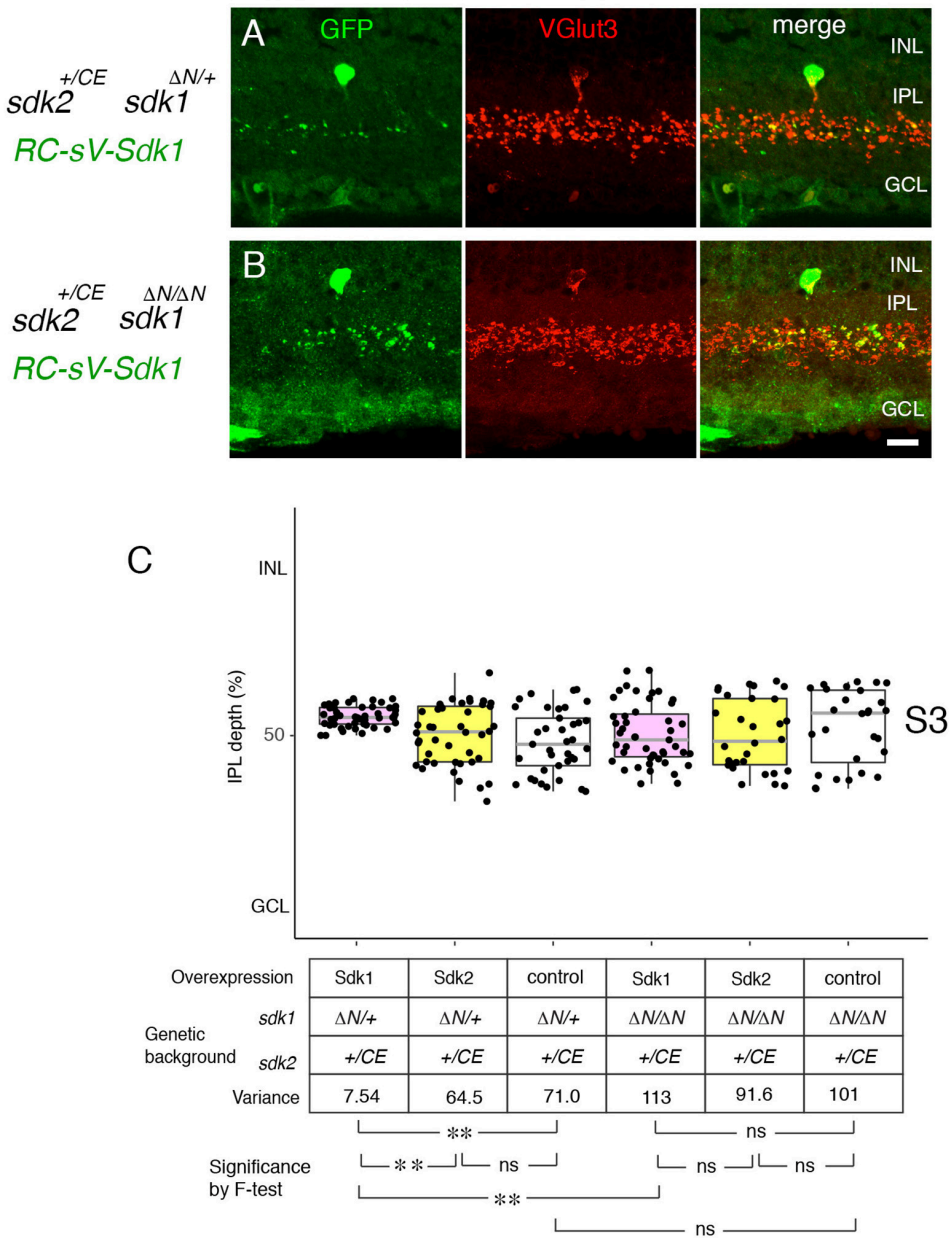
Sdk1 patterns dendrites homophillically. **(A,B)** *Sdk1* was expressed in VG3 cells (*RC-sV-Sdk1;sdk2*^+/*CE*^) in the presence (*sdk1*^+/Δ*N*^) **(A)** or absence (*sdk1* ^Δ*N*/Δ*N*^) **(B)** of endogenous Sdk1. Pups were injected with tamoxifen at P2, and dissected at P32. Narrowing of arbors elicited by ectopic expression of *sdk1* did not occur in the absence of endogenous Sdk1. Bar, 10μm. **(C)** Laminar positioning of GFP+ spots from indicated genotypes was measured in micrographs such as those shown in **(A,B)**, and presented as boxplots as in Figure 5 (*n* = 5 neurons from each of 3 animals). Statistical significance of variance by *F*-test: ns, *F* > 0.1; **F < 0.001.

We then used this model to ask whether Sdk1 acts homophilically to pattern dendritic arbors. To this end, we expressed *sdk1* in VG3-ACs in the absence of endogenous *sdk1* (*RC-vS-Sdk1*; *sdk2-CreER*; *sdk1*^Δ*N*/Δ*N*^ ) (Figure 8). In this case, ectopic Sdk1 had no significant effect on VG3-AC arbors, suggesting that the effects of Sdk1 on dendrites require homophilic interactions among arbors.

### Sensitive Period for Sdk1 Function

**Figure 9:**
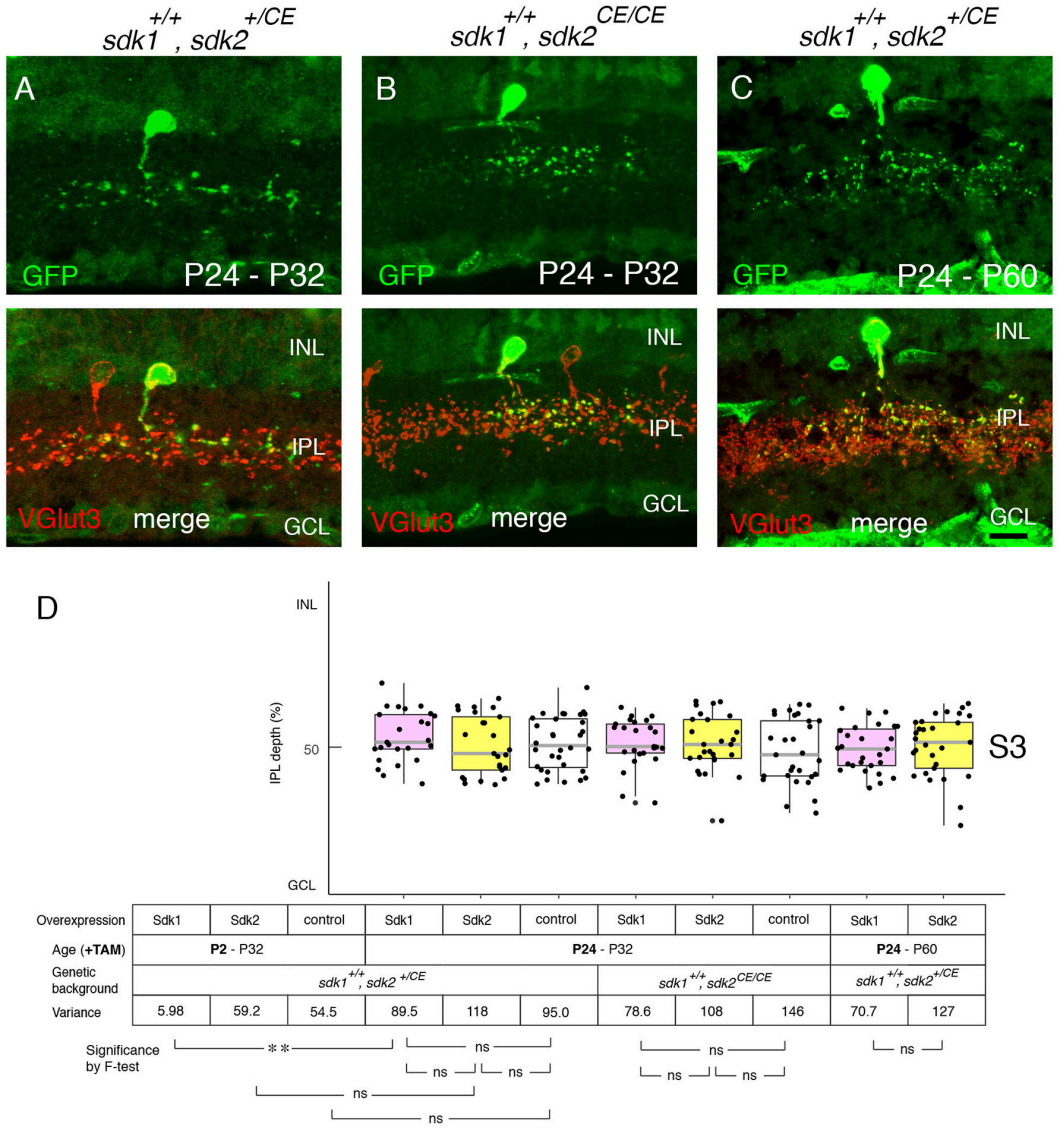
Sensitive period for Sdk1 overexpression. **(A–C)** *RC-sV-Sdk1* was crossed to *sdk2*^+/*CE*^ **(A,C)** or *sdk2*^*CE*/*CE*^ **(B)**. In this experiment, the crosses were carried out as in Figures 6, 7, but pups were injected with tamoxifen at P24 instead of P2, and dissected at P32 **(A,B)** or P60 **(C)**. Overexpression of sdk1 starting at P24 did not affect the morphology of VG3 cells. Bar, 10μm. **(D)** The laminar positioning of GFP+ spots from indicated genotypes was measured in micrographs such as those shown in **(A,C)**, and presented as boxplots as in Figure 5 (*n* = 6 neurons from each of 2–3 animals). Statistical significance of variance by *F*-test: ns, *F* > 0.1; **F < 0.001. The data for P2-P32 are from Figure 5G for comparison.

We next used the ectopic expression model to ask whether Sdk1 can remodel dendrites after dendritic growth is over. To this end, tamoxifen was injected at P24 after IPL sublamination had been established. The morphological change documented above for P2 tamoxifen treatment was not observed at either P32 (Figures 9A,B,D) or P60 (Figures 9C,D). This result suggests that once lamination patterns are established, they are resistant to remodeling by Sdk1.

### Effects of Sdk1 on Starburst Amacrines and J-RGCs

**Figure 10:**
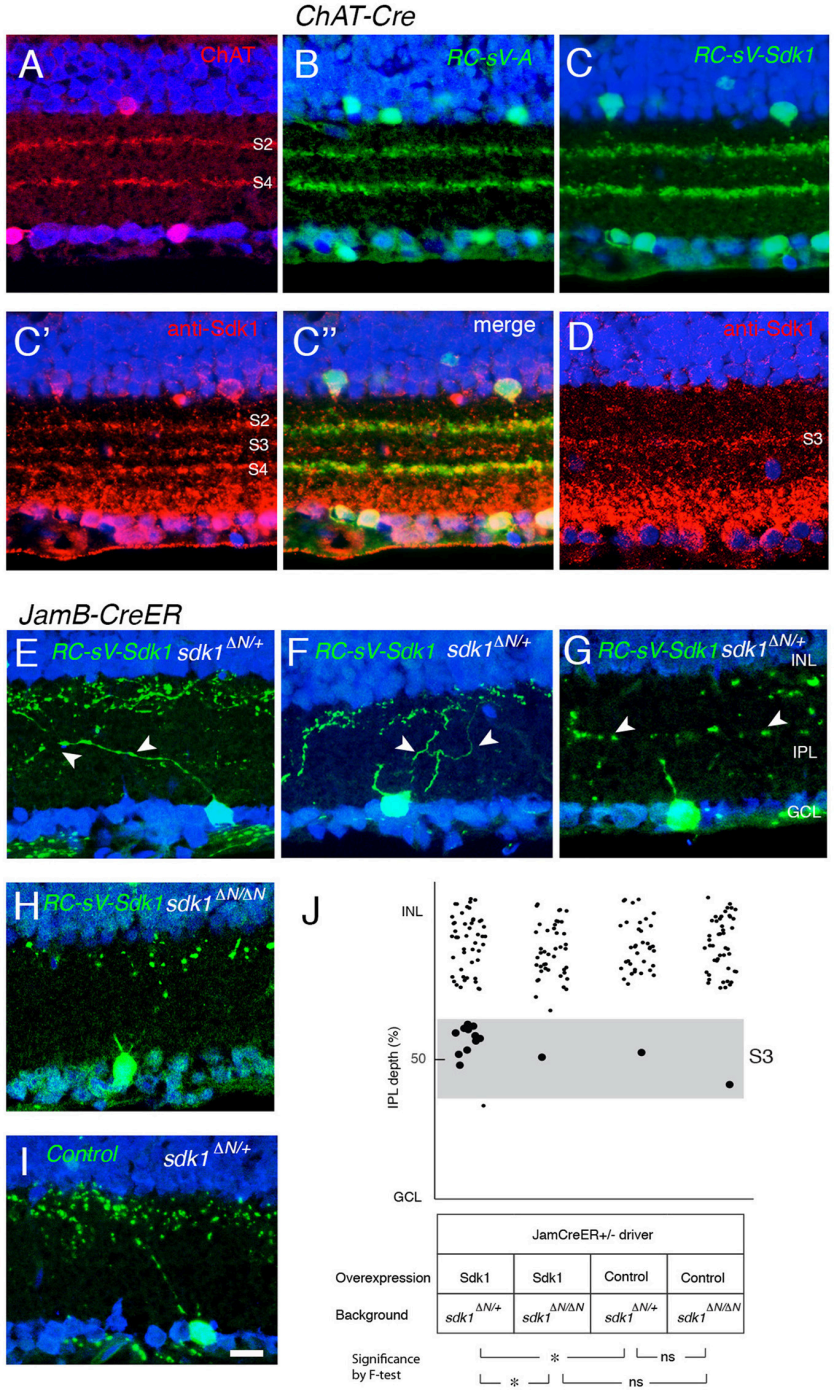
Sdk1 overexpression in starburst amacrine cells and J-RGCs. **(A)** Starburst amacrine cell dendrites stratify in S2 and S4. P30 section stained with anti-ChAT. **(B)** Overexpression of Venus (GFP) in starburst amacrine cells (*RC-sV-A; ChAT-Cre*) labels S2 and S4. **(C)** Overexpression of Sdk1 and Venus (GFP) in starburst amacrine cells (*RC-sV-Sdk1; ChAT-Cre*) labeled tight fascicles in S2 and S4, indistinguishable from controls **(A,B)**. Staining with anti-Sdk1 confirms ectopic expression (C′,C″). **(D)** Immunostaining of wildtype P30 retina with anti-Sdk1 antibodies. Comparison with A′ indicates that recombinant Sdk1 is present at levels similar to those of endogenous Sdk1. **(E–I)** J-RGCs labeled in *JamB*^*CE*^ *;RC-sV-Sdk1; sdk1* ^+/Δ*N*^**(E–G)**, *JamB*^*CE*^ *;RC-sV-Sdk1*; *sdk1* ^Δ*N*/Δ*N*^**(H)** or *JamB1*^*CE*^*;RC-sV-A; ; sdk1* ^+/Δ*N*^ **(I)** mice. Pups were injected with tamoxifen at P2, and dissected at P32. J-RGC dendrites normally arborize in S1 **(I)** but expression of *sdk1* led to formation of ectopic processes in S3 **(E–G)**. Mistargeting arbors elicited by ectopic expression of *sdk1* did not occur in the absence of endogenous Sdk1 (*sdk1* ^Δ*N*/Δ*N*^) **(H)**. Bar, 10μm. **(J)** Laminar position of GFP+ spots from indicated genotypes were plotted as in Figure 5 (*n* = 5 neurons from each of 3 animals). Dots in S3 (40–60% IPL depth) were enlarged for emphasis. Statistical significance of variance by *F*-test: ns, *F* > 0.1; *F < 0.01.

Finally, we asked whether ectopic Sdk1 could affect lamination of other cell types. We tested two types for which reliable cre drivers were available: starburst amacrine cells (*Chat-cre*) (Rossi et al., 2011) and J-RGCs (*JamCreER*) (Kim et al., 2008). Starburst amacrine cell dendrites arborize in two narrow bands in S2 and S4. Levels of ectopically expressed Sdk1 were similar to endogenous levels in S3 (Figures 10A–D). However, the lamination of starburst amacrine cells appeared unaffected by the ectopic expression of *sdk1*.

J-RGC dendrites ascend through the IPL, crossing S3 to arborize in S1. In this case, overexpression of Sdk1 let to formation of dendritic branches in S3 (Figures 10E–J). As was the case for remodeling of VG3-AC dendrites, no remodeling was observed in a *sdk1*^Δ*N*/Δ*N*^ background (Figures 10H,J), indicating that Sdk1 acts homophilically in J-RGCs.

## Discussion

A group of four closely related immunoglobulin superfamily adhesion molecules has been implicated in assembly of neural circuits in chick retina: Sdk1, Sdk2, Dscam and DscamL (Yamagata et al., 2002; Yamagata and Sanes, 2008, 2010, 2012a). Of these, three have also been shown to play roles in assembly of neural circuits in mouse retina: Sidekick 2, Dscam and DscamL (Fuerst et al., 2008, 2009; Krishnaswamy et al., 2015). Here, to complete this set of studies, we investigated the expression and role of Sdk1 in developing retina.

### Sdks Mediate Sublaminar Specificity in Retina

*Sdk1* and *Sdk2* are each expressed by defined types of retinal neurons. Of ~140 total retinal neuronal types in mice, 11 (~7%) express *sdk1* and/or *sdk2* at appreciable levels. Of these, 3 express *sdk1* but not *sdk2*, 5 express *sdk2* but not *sdk1* and 3 express both *sdk1* and *sdk2* (Figure 3K).

Of particular note are two pairs of *sdk*-positive neurons with arbors in S3. VG3-ACs and W3B-RGCs are both *sdk2*-positive and arborize in S3. Likewise, 2CA-ACs and S3-RGCs are both *sdk1*-positive and arborize in S3. The pairs differ, however, in their sublaminar restriction: the Sdk2-positive cells arborize diffusely within S3, whereas the Sdk1-positive cells arborize in a narrow stratum at the center of S3. Moreover, VG3-ACs synapse strongly whereas 2CA-ACs synapse only weakly on W3B-RGCs (Krishnaswamy et al., 2015). Together, these results suggest that the *sdk1*- and *sdk2*-positive pairs comprise separate channels in S3.

Genetic studies indicate that *sdk1* and *sdk2* mutants not only mark these channels but are necessary for their formation. The laminar restriction of VG3-ACs and W3B-RGCs is disrupted in *sdk2* mutants, whereas there is no detectable effect on arbors of 2CA-ACs and Sdk1+S3-RGCs. Conversely, the laminar restriction of 2CA-ACs and Sdk1+S3-RGCs is disrupted in *sdk1* mutants, with no detectable effect on arbors of VG3-ACs and W3B-RGCs. Physiological studies show that functional connectivity of VG3-ACs with W3B-RGCs is dramatically reduced in *sdk2* mutants, with no effect on the weak 2CA-AC to W3B-RGC connectivity. As noted above, we have not yet been able to target Sdk1+S3-RGCs for recording, but we speculate that they will exhibit the opposite pattern—strong synapses from 2CA-ACs, weak synapses from VG3-ACs, loss of connectivity in *sdk1* mutants, and no defects in *sdk2* mutants.

We also found that *sdk1*^+^*sdk2*^+^ interneurons and RGCs share laminar restriction, with rod bipolar cells, ON-α sustained RGCs and M2 intrinsically photosensitive RGCs all of which arborize in S5. In this case, however, we detected no morphological defects in the arbors of these cells in *sdk1* or *sdk2* mutants or in *sdk1sdk2* double mutants. Rod bipolars also express the related recognition molecule, DscamL (Fuerst et al., 2009), and it possible that deletion of both recognition systems would be required to disrupt these arbors.

Cells that express both *sdk1* and *sdk2* can, of course, interact with cells that express either *sdk1*^+^ or *sdk2*^+^, generating bifurcated circuits. In this regard, it is intriguing that Sdk1-positive A17 amacrine cells also arborize extensively in S5. A circuit involving A17 amacrine cells has been characterized in rabbits, and A17 terminals contact both rod bipolar axons and ON-RGC dendrites (Diamond, 2017). Likewise, Sdk2-positive horizontal cells interact with rod bipolar dendrites in the outer plexiform layer.

### Sdk1 Acts Instructively and Homophilically During Arbor Formation

To analyze the mechanism by which Sdk1 acts we used a gain-of-function strategy, expressing it in cells that are normally *sdk1*-negative. From our results, we draw three conclusions. First, Sdk1 acts instructively. When expressed in VG3-ACs, it remodels their arbors in S3 from their normal diffuse pattern to the narrow, central pattern characteristic of Sdk1-positive 2CA-ACs. Likewise, expression of Sdk1 in J-RGCs, which arborize in S1, results in formation of ectopic branches in S3. Sdk1 is restricted in its potency, however; expression in starburst amacrines, with dendrites that border S3, has no detectable effect. We do not know what cell type-specific factors affect the ability of Sdk1 to pattern arbors.

Second, Sdk1 acts homophilically in that its ability to pattern arbors require that it be expressed in neighboring cells. Thus, expression of *sdk1* in small numbers of VG3-ACs or J-RGCs fails to affect their arbors in a *sdk1* mutant background. The simplest interpretation of this finding is that ectopically expressed *sdk1* leads to fasciculation of neurites with those of cells that express *sdk1* endogenously. This mechanism is consistent with the observation that formation of synapses between *sdk2*-positive cells requires expression of *sdk2* on both synaptic partners (Krishnaswamy et al., 2015).

Third, Sdk1 acts during a restricted period of development, as arbors are forming. When ectopic expression is initiated after arbors have already formed, it has no detectable effect over a period of at least 1 month. Apparently, once arbors have matured, they become resistant to remodeling.

Taken together, we and Fuerst, Burgess, and colleagues have now demonstrated roles for Sdk1, Sdk2, Dscam and DscamL1 in patterning the IPL in both chick and mouse retina (Yamagata et al., 2002; Fuerst et al., 2008, 2009, 2012; Yamagata and Sanes, 2008, 2010; Krishnaswamy et al., 2015; Garrett et al., 2016). There are interesting differences in phenotype among molecules and between species. For example, Dscam and DscamL1 appear to act by an inhibitory mechanism in mice but an attractive mechanism in chicks, whereas Sdks act by an attractive mechanism in both species (Fuerst et al., 2008, 2009; Yamagata and Sanes, 2008; and this paper). Also, the four relatives are expressed by almost entirely non-overlapping populations in chick retina, whereas some retinal neuronal types express both *sdks*, and/or *sdks* and *dscams* (Fuerst et al., 2008, 2009 and this paper). Despite these differences, however, results to date make a strong case that selective expression of these recognition molecules, as well as the closely related contactins (Yamagata and Sanes, 2012a; Peng et al., 2017), generates an “immunoglobulin superfamily code” critical for synaptic specificity in the vertebrate retina.

## Author Contributions

MY and JS planned experiments and wrote the manuscript. MY performed experiments and analyzed data.

### Conflict of Interest Statement

The authors declare that the research was conducted in the absence of any commercial or financial relationships that could be construed as a potential conflict of interest.
