# Supplementary material for: Expression and Roles of the Immunoglobulin Superfamily Recognition Molecule Sidekick1 in Mouse Retina
Source: Front Mol Neurosci. 2019 Jan 9;11:485. doi: 10.3389/fnmol.2018.00485 (PMC6333872; doi:10.3389/fnmol.2018.00485)
Supplement: Supplementary file 1 [file Data_Sheet_1.PDF]

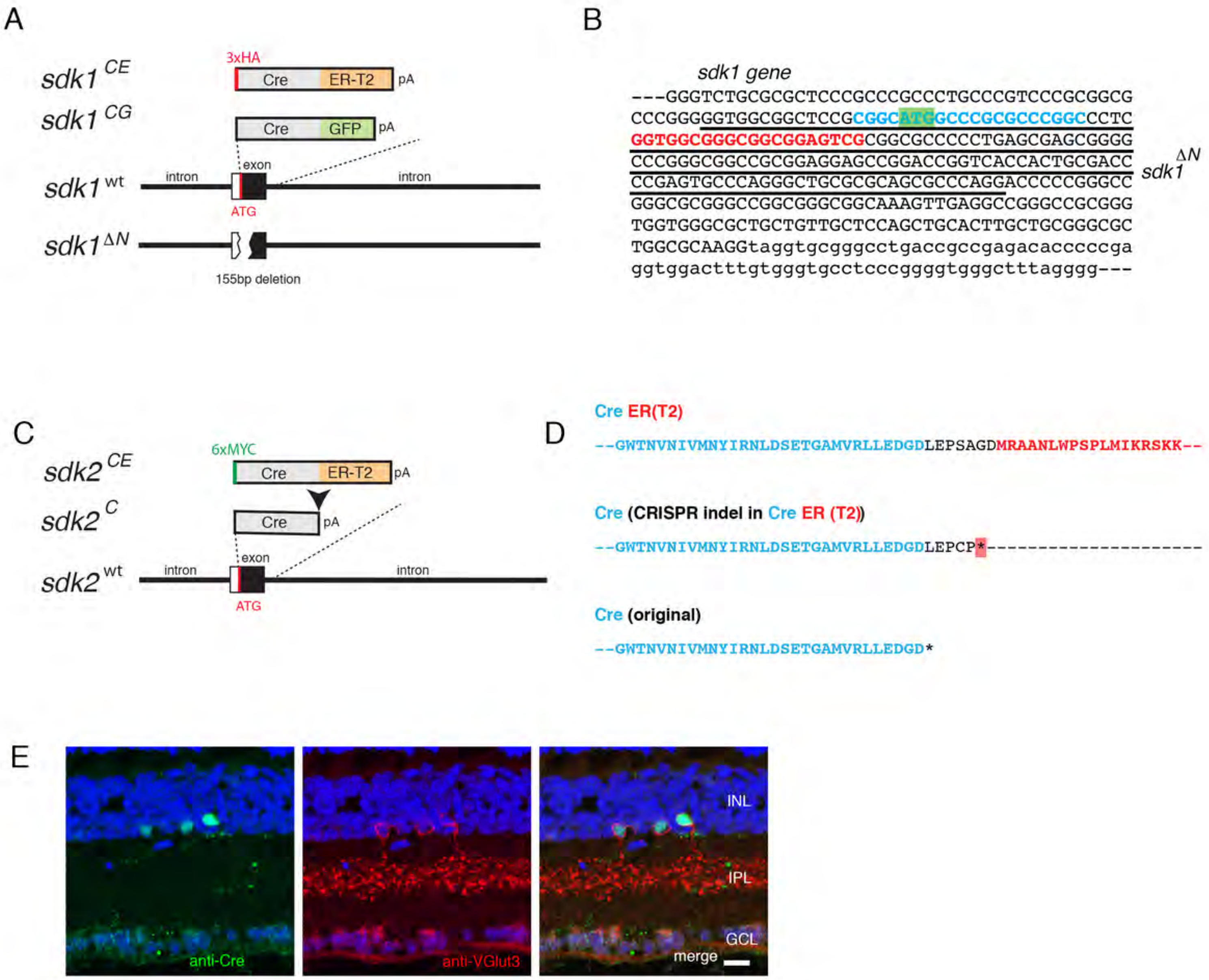

Figure S1. Sdk1 and Sdk2 mutant alleles.

(A) *Sdk1* alleles. In $sdk1^{CE}$, a haemagglutinin (HA) tagged CreER^{T2} replaced the first coding exon. $sdk1^{CG}$ was generated by inserting CreGFP at the same sequence. $sdk1^{\Delta N}$ has a 155bp deletion (resulting in 46 aa deletion of Sdk1 protein) around the initiation codon.

(B) Deletion in the $sdk1^{\Delta N}$ allele. Underlined nucleotides were deleted. Blue and red letters indicate target sequences for sgRNAs.

(C) *Sdk2* alleles. $Sdk2^{CE}$ was generated in the same way as $sdk1^{CE}$ but the CreER^{T2} was fused to a Myc epitope tag. $sdk2^{C}$ was derived from $sdk2^{CE}$ by CRISPR-mediated generation of indels at the junction of Cre and ER^{T2}.

(D) Sequence of the truncated Cre in the $sdk2^{C}$ allele. Deletion at the junction between Cre (blue) and ER^{T2}(red) sequences in $sdk2^{CE}$ leads to truncation in the linker (black).

(E) Expression of Cre in $sdk2^{+/C}$ mice as revealed by anti-Cre antibody. Nuclei of VG3 amacrine cells are stained.

Bar, 10µm.

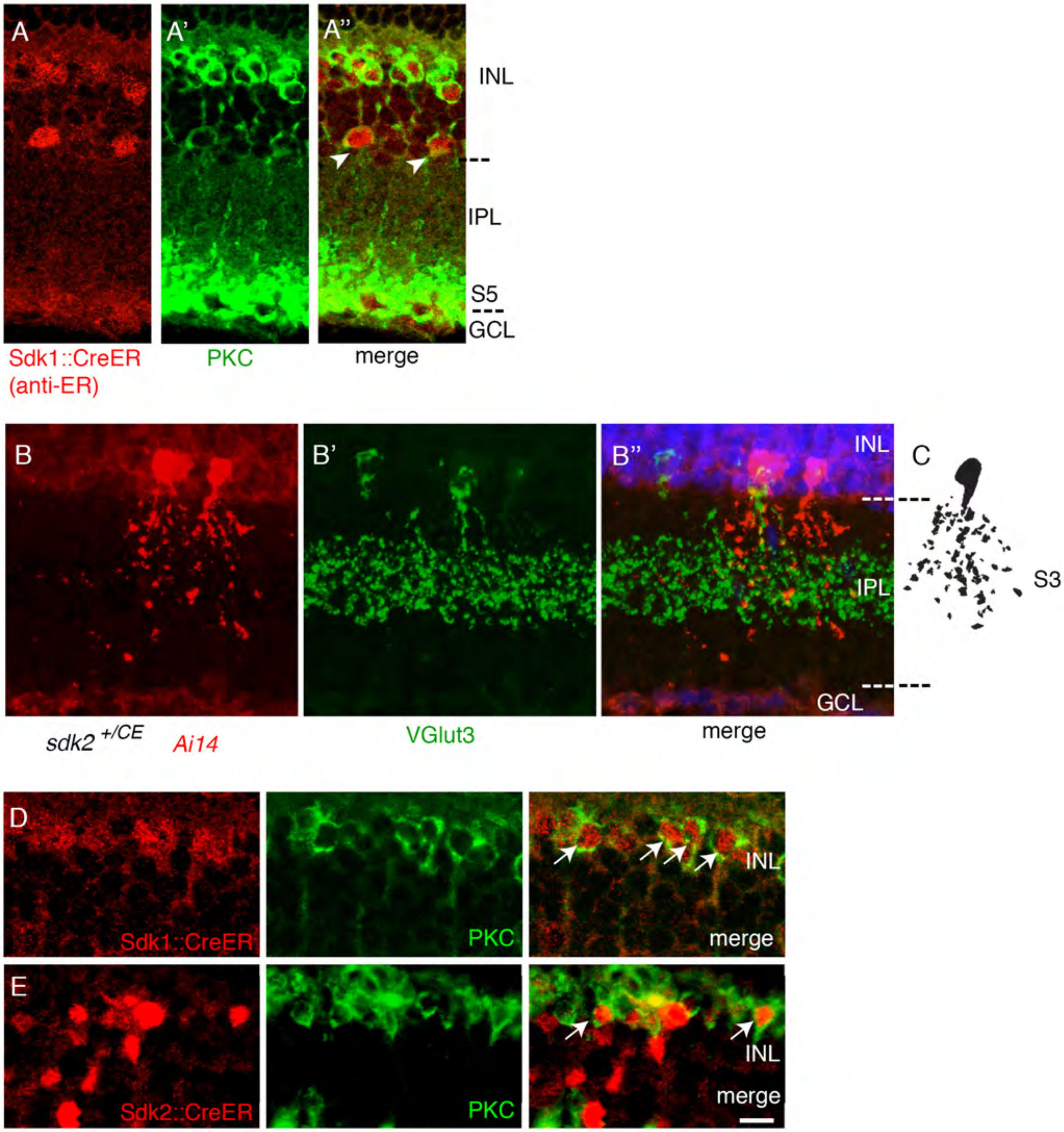

Figure S2. Amacrine cell types expressing *sdk1* or *sdk2*.

(A) Section from a P30 sdk1$^{+/CE}$ retina was stained with anti-ER (A) and anti-PKCα(A'). Sdk1 is present in PKCα+ rod bipolar cells (also see Figure 3) and also in a set of PKCα+ amacrine cells (arrowheads). These are not 2CA cells, which are PKCα-negative. Because A17 amacrine cells are PKCα+ in rat (Puthussery and Fletcher, 2007; Downie et al., 2009), this result supports the idea that mouse A17 cells are Sdk1+.

(B,C) Section from a *sdk2*$^{+/CE}$;*Ai14* retina stained with anti-VGlut3. In addition to the VG3 amacrine cells, a small number of VGlut3-negative narrow field amarines that arborize in S1-4 is *sdk2*-positive. This cell type is negative for Dab1 and PKCα (not shown), excluding the possibility of their being AII or A17 amacrine cells. One such cell is traced in C.

(D) Double staining of a *sdk1*$^{+/CE}$ retinal section with anti-ER and anti-PKCα (PKC), which marks RB (arrows; Greferath et al., 1990).

(E) Double staining of a *sdk2*$^{+/CE}$ retinal section with anti-ER, and anti-PKCα (arrows). Bar, 10 µm.
