# Supplementary material for: Expression and Roles of the Immunoglobulin Superfamily Recognition Molecule Sidekick1 in Mouse Retina
Source: Front Mol Neurosci. 2019 Jan 9;11:485. doi: 10.3389/fnmol.2018.00485 (PMC6333872; doi:10.3389/fnmol.2018.00485)
Supplement: Supplementary file 2 [file Data_Sheet_2.PDF]

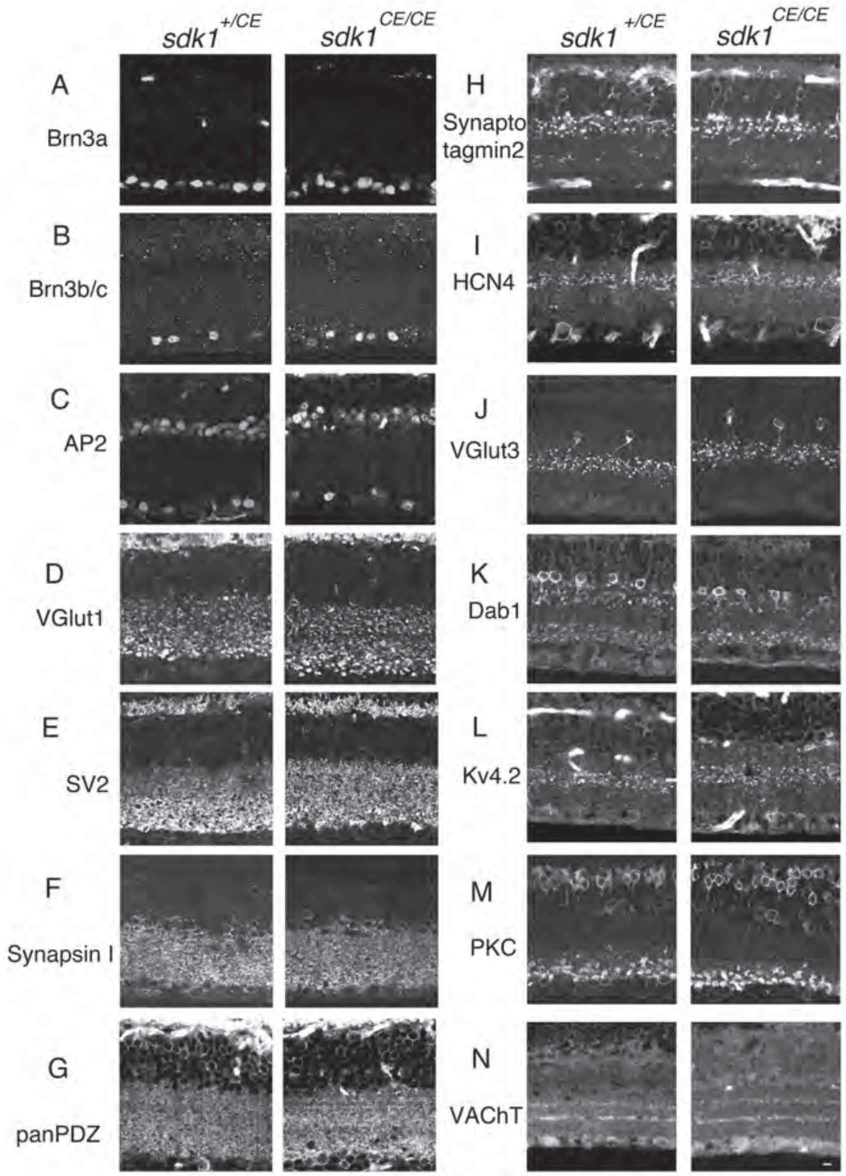

Figure S3. Molecular architecture of retina is unaffected in *sdk1* mutants

Each pair of micrographs shows sections from $sdk1^{+/CE}$ retina (left) and $sdk1^{CE/CE}$ retina (right) stained with antibodies to the indicated marker. In no case do levels or patterns of immunoreactivity differ detectable between genotypes.

(A, B) Brn3a and Brn3b/c are expressed by overlapping subsets of RGCs. (C) AP2 is expressed by most amacrine cells. (D) VGlut1 is present in terminals of photorecetoprs and bipolar cells in the OPL and IPL, respectively. (E,F) SV2, and synapsin I, components of synaptic vesicles, are present in nerve terminals of many cell types in the OPL and IPL. (G) Antibody Pan-PDZ labels postsynaptic density proteins of the PSD95 family. (H) Synaptotagmin2 is present in terminals of type 2 and 6 bipolar cells. (I) HCN4 is concentrated in terminals of type 3A bipolar cells. (J) VGlut3 is expressed by VG3 amacrine cells; protein marks both somata in the INL and terminals in the IPL. (K) Dab1 is expressed by AII amacrine cells; protein marks both somata in the INL and terminals in the IPL. (L) Kv4.2 is concentrated in terminals of a subset of RGCs. (M) PKCα is expressed by rod bipolar cells and a subset of amacrine cells; protein marks both somata in the INL and terminals in the IPL (also see Figures 6F-I). (N) VAChT is concentrated in dendrites of starburst amacrine cells in the IPL. Bar, 10 µm.

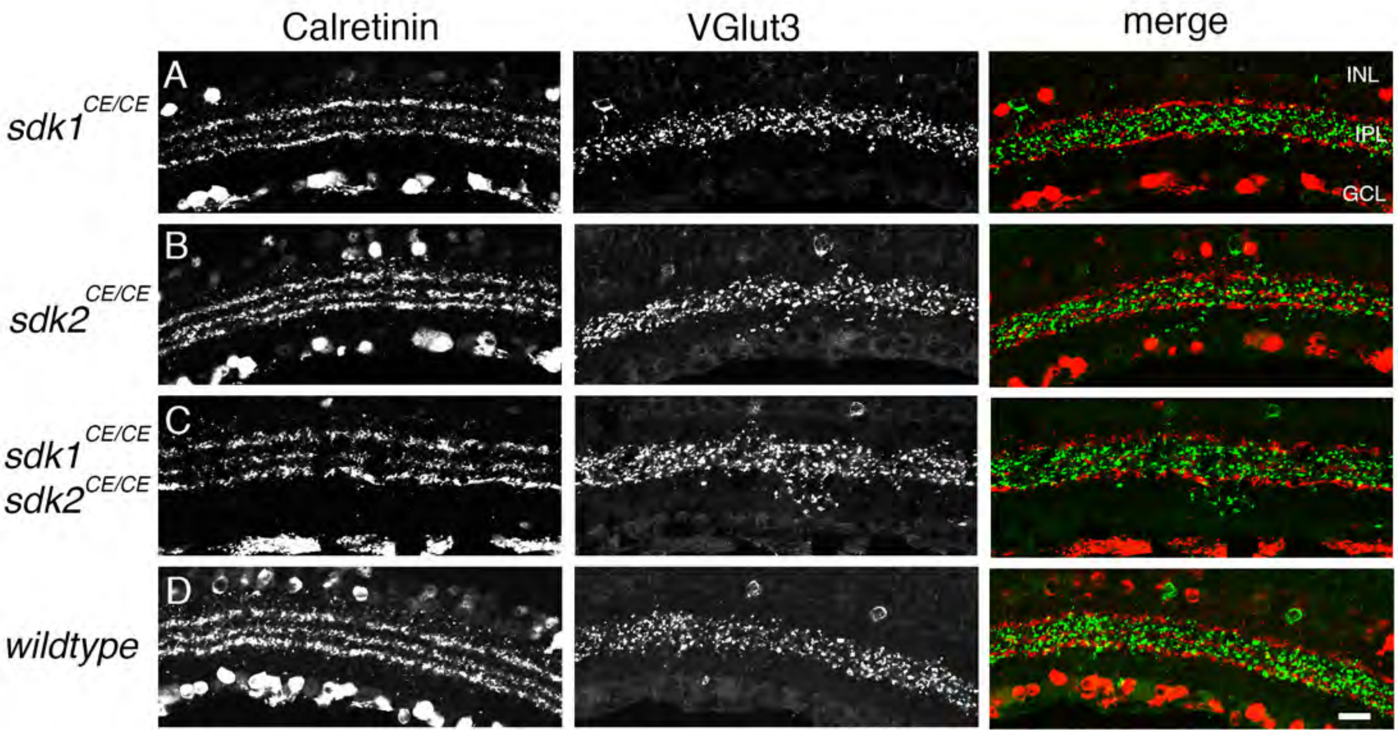

Figure S4. VGlut3 phenotype in sdk1, sdk2, and double knock-out retina.

(A-D) Calretinin-positive processes arborize more diffusely in *sdk1*$^{CE/CE}$ retina than in controls. In contrast, VGlut3-positive processes more diffusely in *sdk2*$^{CE/CE}$ mutants than in contols. Both of these phenotypes are observed, but not enhanced in *sdk1*$^{CE/CE}$ / *sdk2*$^{CE/CE}$ double mutants. Bar, 10 μm.
